# Suction versus traditional ureteral access sheaths in retrograde intrarenal surgery: an updated systematic review and meta-analysis of stone-free rates and complications

**DOI:** 10.1007/s00345-026-06873-5

**Published:** 2026-11-05

**Authors:** José Arnaldo Shiomi da Cruz, Bruno D. Terada, Felipe G. A. Gonçalves, Leonardo O. D. da Silva, Artur S. Almeida, Breno C. Porto, Rodrigo A. S. Sardenberg, Carlo C. Passerotti, Eugenio Vicari-Neto, Gustavo Freschi, Diego P. Resuto, Alex E. Meller, Renato N. Pedro, Antonio C. Lopes Neto, Renato Fraietta, Fernando G. de Almeida, Wilson R. Molina, Ronaldo S. Maia

**Affiliations:** 1 Clinical Research Institute - Hapvida NotreDame, São Paulo, Brazil; 2 German Hospital Oswaldo Cruz, São Paulo, SP Brazil; 3 Department of Urology, Federal University of São Paulo (UNIFESP), 740 Botucatu Street, 5th Floor, Suite 504, Octávio de Carvalho Building, Vila Clementino, São Paulo, 04023-900 SP Brazil; 4 São Leopoldo Mandic School of Medicine, Campinas, SP Brazil; 5 School of Medical Sciences, State University of Campinas (UNICAMP), Campinas, SP Brasil; 6 Department of Urology, ABC Medical School, Santo André, SP Brazil; 7 Department of Urology, The University of Kansas Health System, Kansas, KS USA; 8 Department of Urology, Ninth of July University, São Bernardo do Campo, SP Brazil

**Keywords:** Meta-analysis, Nephrolithiasis, Ureteroscopy, Suction, Ureteral access sheath

## Abstract

**Purpose:**

Suction ureteral access sheaths (S-UAS), fixed-tip or flexible and navigable (FANS), are proposed to improve stone clearance and reduce infections in retrograde intrarenal surgery (RIRS); we updated our 2025 meta-analysis and asked whether tip design changes the effect.

**Methods:**

Five databases were searched from inception to March 2026 for comparative studies of S-UAS versus traditional sheaths (T-UAS) in adult RIRS (PROSPERO CRD420251164154). Outcomes were residual fragments at early (up to 3 weeks) and late (4 weeks or later) assessment, fever, sepsis, septic shock, severe complications, operative time and hospital stay, analyzed with random-effects models, subgroups by design and tip design, small-study tests and GRADE.

**Results:**

Twenty-nine studies (four randomized; 5,604 patients) were included. S-UAS reduced residual fragments at early (RR 0.49; 95% CI 0.42–0.58; 22 studies) and late assessment (RR 0.53; 0.45–0.63; 23 studies), fever (RR 0.43; 0.34–0.53), sepsis (RR 0.47; 0.31–0.72) and severe complications (RR 0.48; 0.33–0.69). Septic shock was rare (4 versus 12 events; RR 0.41; 0.15–1.15). Operative time did not differ; hospital stay was 0.32 days shorter, but not robustly so. Post hoc, the late benefit was larger with tip-flexible (RR 0.47) than with fixed-tip sheaths (RR 0.72; *p* = 0.03). Certainty was moderate for stone clearance and fever (low for early clearance in non-randomized studies), otherwise low or very low.

**Conclusion:**

S-UAS probably improve stone clearance and reduce fever and may reduce sepsis; the evidence on severe complications is very uncertain. The effect on septic shock and a possible advantage of the flexible tip need randomized confirmation.

**Supplementary Information:**

The online version contains supplementary material available at https://doi.org/10.1007/s00345-026-06873-5.

## Introduction

Nephrolithiasis affects approximately 10% of the population [1, 2], and flexible ureteroscopic lithotripsy has become a standard treatment for renal stones [3–5]. The ureteral access sheath (UAS) facilitates instrument passage and fluid drainage during retrograde intrarenal surgery (RIRS) [6–8]. Suction ureteral access sheaths (S-UAS) add an aspiration channel that removes fragments and irrigation fluid during lithotripsy and limits the rise in intrarenal pressure. The first generation of these devices has a fixed tip positioned at the pyeloureteral junction, whereas the current generation, the flexible and navigable suction sheath (FANS), has a bendable distal segment that follows the ureteroscope into the calyces [9–12].

Guideline recognition is still partial. The American Urological Association has issued a conditional recommendation stating that clinicians may choose a flexible and navigable suction UAS when a sheath is used during ureteroscopy, whereas the European Association of Urology has not yet taken a position on the device [13–16]. In 2025, our group published a meta-analysis of eight comparative studies that favored S-UAS for stone clearance, fever and overall complications, with the caveat that the evidence was small and came mostly from Chinese centers [17]. Since that search, in August 2024, more than twenty new comparative studies, three of them randomized, have been published, sheaths of both tip designs have been compared with traditional sheaths under the same label, and at least ten further syntheses have appeared, none of them separating the two designs among suction-versus-no-suction comparisons [10, 12, 18–27]. Cochrane guidance recommends updating a review when the new evidence is likely to change the precision or the credibility of its conclusions [28], and both conditions were met here.

We therefore updated the systematic review and meta-analysis comparing S-UAS with traditional ureteral access sheaths (T-UAS) in RIRS, with three additions to the previous synthesis: a subgroup analysis by tip design, a formal assessment of small-study effects, and a summary of the certainty of evidence for each outcome.

## Materials and methods

This systematic review and meta-analysis was conducted and reported in accordance with the Cochrane Handbook for Systematic Reviews of Interventions and the Preferred Reporting Items for Systematic Reviews and Meta-Analyses (PRISMA) 2020 statement [29]. The protocol was registered in PROSPERO (CRD420251164154). Six departures from the registered protocol are declared: the intervention was defined explicitly as any suction sheath, with a fixed or a flexible tip, once the included devices had been re-examined, an explicit exclusion of studies with a suction comparator was added, operative time and hospital stay were accepted as qualifying outcomes, the two time points of stone-free assessment were redefined as up to 3 weeks and 4 weeks or later to match the schedules of the included studies, the complication outcome was renamed severe complications and given an operational definition, and the subgroup analysis by tip design and the small-study tests were added post hoc.

### Search strategy

MEDLINE (via PubMed), Embase, Scopus, Web of Science and the Cochrane Library were searched from database inception to March 2026. The strategy combined controlled vocabulary and free-text terms for suction or aspiration, ureteral access sheath, and retrograde intrarenal surgery. The MEDLINE strategy was: (Perfusion OR suction* OR suctioning OR aspirat* OR vacuum OR “negative pressure” OR “pressure control” OR evacu* OR tip-bendable OR “tip flexible” OR “flexible tip” OR tip-flexible) AND ((“ureteral access sheath“[tiab]) OR (“ureteral sheath“[tiab]) OR (“ureteral access“[tiab]) OR (“suctioning ureteral access sheath“[tiab]) OR (UAS[tiab]) OR (FANS[tiab]) OR (S-UAS[tiab]) OR (“negative pressure“[tiab] AND “ureteral access sheath“[tiab]) OR (vacuum[tiab] AND “ureteral access sheath“[tiab]) OR ((fluid[tiab] OR irrigation[tiab]) AND (aspirat*[tiab] OR suction*[tiab]) AND “ureteral access sheath“[tiab])) AND ((“retrograde intrarenal surgery“[tiab]) OR (RIRS[tiab]) OR (“flexible ureteroscopy“[tiab]) OR (“flexible ureteroscope“[tiab]) OR (“digital flexible ureteroscopy“[tiab]) OR (ureterorenoscopy[tiab]) OR (“ureteroscopic lithotripsy“[tiab]) OR (“ureteral stones“[tiab]) OR (“kidney stones“[tiab]) OR (urolithiasis[tiab]) OR (“renal calculi“[tiab])). The same terms were adapted to the syntax of the other databases (Supplementary Table 1). The reference lists of the included studies and of the previous systematic reviews on the subject were screened by hand.

### Eligibility criteria

Eligibility followed the PICOS framework. Population: adults (18 years or older) undergoing RIRS for single or multiple renal or proximal ureteral stones. Intervention: any ureteral access sheath with an integrated aspiration channel connected to active suction (S-UAS), whether the distal segment was flexible (FANS) or fixed. Comparator: a traditional ureteral access sheath without a suction mechanism or any other device-integrated system for intrarenal pressure control (T-UAS). Outcomes: at least one of stone-free status, postoperative fever, sepsis, septic shock, postoperative complications, operative time or length of hospital stay. Study design: randomized controlled trials (RCTs) and non-randomized comparative studies (non-RCTs), whether prospective, retrospective or matched.

Studies were excluded if they (1) included patients younger than 18 years; (2) lacked a comparator group; (3) enrolled patients with active urinary tract infection at the time of surgery; (4) were restricted to stones larger than 3 cm or to staghorn calculi; (5) were restricted to patients with relevant anatomical abnormalities such as solitary, horseshoe or transplanted kidneys; (6) were restricted to bilateral procedures or to surgery performed concurrently with RIRS; (7) connected the comparator sheath to a suction device, so that both arms received aspiration; or (8) presented overlapping cohorts, in which case the study with the largest dataset was retained. Studies in which a minority of patients fell outside criteria 4 to 6 were retained and examined in a sensitivity analysis. Case reports, reviews and meeting abstracts were excluded. Overlap between cohorts was checked by author, center and enrollment period.

### Screening and selection process

After duplicate removal with EndNote online 20 (Clarivate, Philadelphia, PA) [30], two independent reviewers (B.D.T. and A.S.A.) screened titles and abstracts, and disagreements were resolved by a third researcher (B.C.P.). Full texts were then assessed for eligibility. No automation tools were used for screening. Each included report was re-read to confirm the design of the sheath used in each arm and the absence of suction in the comparator arm.

### Endpoints

The primary endpoint was stone-free status, defined in most studies as the absence of residual fragments larger than 2 to 4 mm on imaging (non-contrast computed tomography, kidney-ureter-bladder radiography or ultrasonography). It was analyzed at two time points, as the included studies scheduled them: early assessment, up to 3 weeks after surgery and on the first postoperative day in most studies, and late assessment, at 4 weeks (28 to 30 days) or later, in most studies at 1 to 3 months. Secondary endpoints were postoperative fever, sepsis and septic shock as defined and counted by the primary authors (Sepsis-3, qSOFA, SIRS or unspecified criteria; Supplementary Table 2), severe postoperative complications, operative time and postoperative length of hospital stay (LOS). Severe complications were defined operationally: events graded III or higher by the Clavien-Dindo classification [31] in the studies that reported grades per event, and, in the studies that did not, events requiring endoscopic or surgical reintervention or intensive care, together with systemic infectious complications reported as sepsis, systemic inflammatory response syndrome or septic shock. Because this construct mixes graded and derived events, the analysis restricted to graded studies is reported alongside it.

### Data extraction

Two authors (B.D.T. and A.S.A.) independently extracted study design, country and enrollment period, patient demographics, stone characteristics and clinical outcomes with a predefined form. The brand and tip design of the suction sheath, the negative pressure applied, the calibre of the sheath in each arm, the proportion of pre-stented patients, previous stone episodes or stone surgery, and the timing, imaging modality and threshold of the stone-free definition were then extracted from each report with verbatim supporting quotations and checked by the authors (Supplementary Table 2). Tip design was classified as flexible when the primary authors described a bendable or navigable distal segment; as fixed when the authors described a sheath kept at the pyeloureteral junction without tip deflection, or when the only device named was the ClearPetra sheath, whose original model has a fixed tip, and no deflection was described; and as mixed when devices of both designs were pooled in one arm without description of the tip. Because three studies named the ClearPetra sheath while describing a bendable tip, and two pooled it with a tip-flexible device, a second classification by brand, which moves the former to the fixed-tip group and the latter to the mixed group, was run as a sensitivity analysis. Where studies reported medians, means and standard deviations were estimated from the median and the interquartile range or the range with the method of Wan et al. [32]; for one study that reported means with ranges, the standard deviations were estimated from the ranges. Two studies that reported hospital stay without any measure of dispersion, or with an interquartile range of zero in both arms, could not be pooled for that outcome. The studies entered from medians were removed in a sensitivity analysis of both continuous outcomes. Where the number of patients assessed for stone-free status was not reported, all treated patients were used as the denominator.

### Quality assessment

Risk of bias was assessed with RoB 2 for randomized trials and ROBINS-I for non-randomized studies [33, 34]. Two authors (F.G.A.G. and L.O.D.S.) performed the assessments independently, with disagreements resolved by consensus. The certainty of evidence was assessed with GRADE [35] for seven outcomes, separately for RCTs and non-RCTs, with one set of rules for both designs: evidence assessed with ROBINS-I starts at high certainty [36]; risk of bias was rated serious when studies at low risk contributed less than half of the weight; inconsistency when I² exceeded 50%, and very serious when it exceeded 90% with point estimates on both sides of the null; indirectness when the outcome was an operational construct; and imprecision when fewer than 300 events were pooled or the confidence interval included both benefit and harm, and very serious when fewer than 20 events were pooled.

### Statistical analysis

Continuous outcomes were summarized as mean differences (MD) and dichotomous outcomes as risk ratios (RR), with 95% confidence intervals (CI). Random-effects models were used throughout, with inverse-variance weighting, restricted maximum likelihood estimation of the between-study variance and a continuity correction of 0.5 for arms with no events; comparisons with no events in either arm contribute to the study and patient counts but not to the pooled estimate. For severe complications, an outcome with many zero-event studies, Mantel-Haenszel weighting with the DerSimonian-Laird estimator was used because the iterative estimator did not converge, and the same model was run for sepsis and septic shock as a check. Prediction intervals were calculated to convey the range of effects expected in future settings. Heterogeneity was quantified with I² and its 95% CI, Cochran’s Q and τ², and explored with leave-one-out analyses and Baujat plots when I² exceeded 50% [37]. Stone-related outcomes were expressed as residual fragment rates (non-stone-free status), so that an RR below 1 favors S-UAS for every outcome. Forest plots were stratified by study design, and a second stratification by tip design was added; between-subgroup differences were tested with the Q statistic, and for tip design the test contrasted the flexible and fixed groups only. Small-study effects were assessed for outcomes with at least ten informative studies by contour-enhanced funnel plots [38], Harbord’s test for binary outcomes, with Peters’ test as a check for rare events, and Egger’s test for continuous outcomes, complemented by trim-and-fill [39–42]. Sensitivity analyses re-included the two studies excluded for suction in the comparator arm, excluded the studies in which a minority of patients fell outside the eligibility criteria, excluded the studies at serious risk of bias, restricted severe complications to graded studies and to studies whose count did not derive from infectious events, restricted operative time and LOS to studies reporting means, and restricted every analysis to tip-flexible sheaths. A two-sided p value below 0.05 was considered significant. The submitted version was analyzed in R 4.6.0 and the revised version in R 4.5.3 with the meta package version 8.5 [43, 44].

## Results

### Study selection and characteristics

**Fig. 1:**
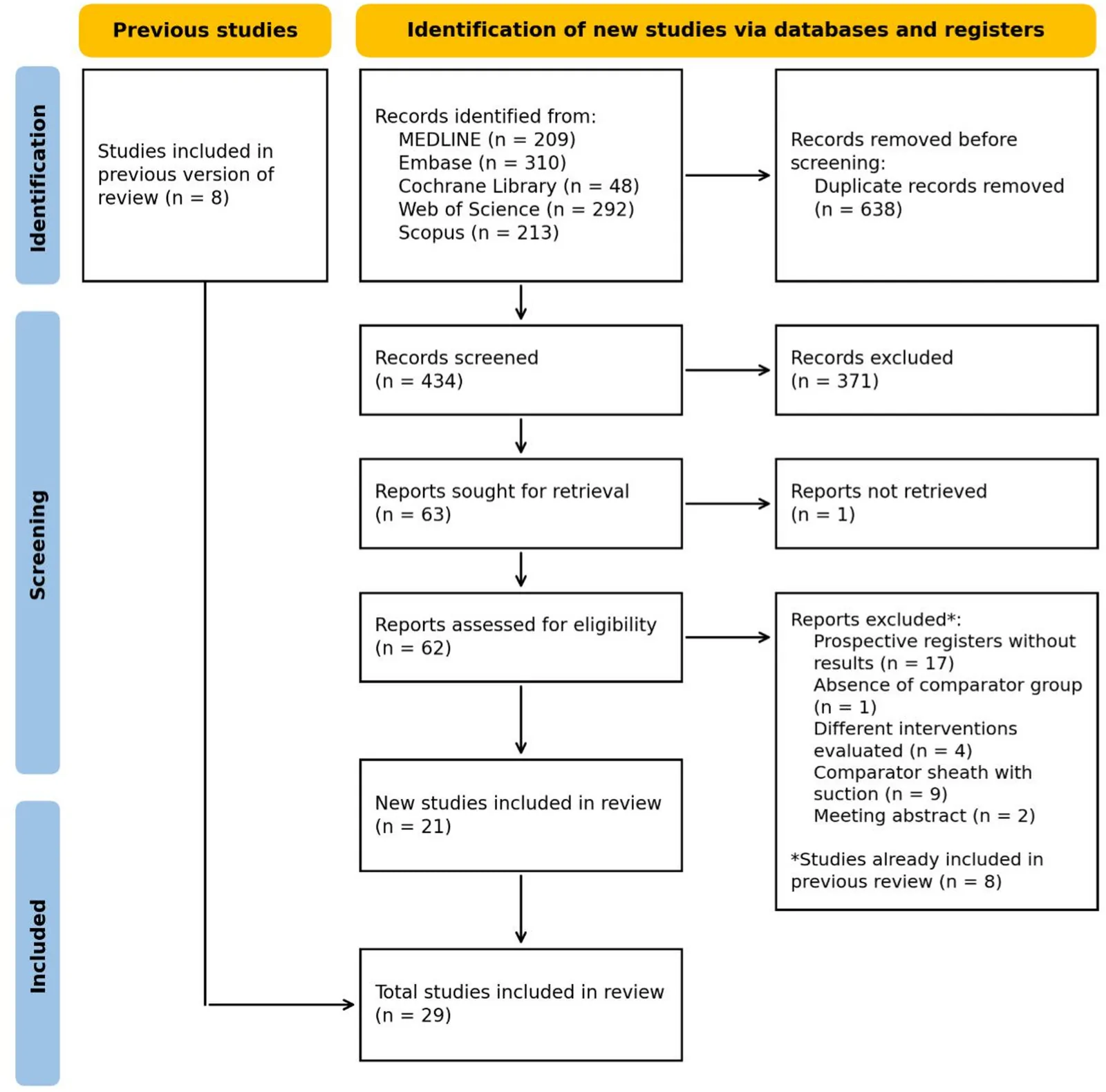
PRISMA 2020 flow diagram of the updated review

The search identified 1,072 records, 434 remained after duplicate removal, 62 reports were read in full and 29 studies met the eligibility criteria [45–73] (Fig. 1). Nine reports compared two suction sheaths and were excluded, among them two that compared a tip-flexible sheath with a fixed-tip sheath also connected to suction [74, 75]. No overlapping cohorts were found; the two Turkish series that share a center enrolled in different periods [47, 62]. The included studies comprised 4 RCTs [45, 49, 57, 73] and 25 non-randomized comparative studies, with 2,600 patients treated with S-UAS and 3,004 with T-UAS; one study reported two strata by stone size, which were entered as separate comparisons [52]. Sixteen studies came from China, four from Turkey, two from Taiwan, one each from Egypt, Italy, Romania, Argentina and the United Kingdom, and two were multinational. Six studies included a minority of patients outside the eligibility criteria (stones larger than 3 cm, staghorn stones or bilateral procedures) [48, 50, 51, 63, 70, 73]. Patient demographics are summarized in Table 1, clinical outcomes in Table 2, and stone size and device characteristics in Supplementary Table 2.

**Table 1:** Design, country and patient characteristics of the included studies

Study (author, year)	Design	Country	Patients, *n*		Male sex, *n* (%)		Age, years		BMI, kg/m²	
			S-UAS	T-UAS	S-UAS	T-UAS	S-UAS	T-UAS	S-UAS	T-UAS
Alhefnawy, 2026	RCT	Egypt	73	72	41 (56.2%)	46 (63.9%)	40 ± 10	41 ± 11	NS	NS
Anil, 2026	Retrospective cohort	Turkey	90	90	62 (68.9%)	66 (73.3%)	48.7 ± 14.7	48.2 ± 14.3	28.8 ± 4.8	28.3 ± 4.6
Arikan, 2025	Retrospective cohort	Turkey	105	102	64 (61.0%)	58 (56.9%)	46.58 ± 15.03	49.1 ± 15.04	NS	NS
Barbour, 2025	Prospective cohort with historical control	United Kingdom	50	50	29 (58.0%)	27 (54.0%)	67 (56–74)*	66 (51–73)*	NS	NS
Cacciatore, 2025	RCT	Italy	62	70	NS	NS	63.3 (48.7–71.5)*	57.1 (47.5–68.3)*	23.9 (22.8–26.1)*	24.8 (23.2–26.4)*
Chen, 2024	Retrospective cohort	China	125	113	76 (60.8%)	65 (57.5%)	45.62 ± 12.93	46.35 ± 14.88	25.21 ± 3.77	25.66 ± 4.35
Chiang, 2025	Retrospective cohort	Taiwan	51	53	30 (58.8%)	38 (71.7%)	61 ± 12.21	57.30 ± 13.82	26.29 ± 3.98	26.74 ± 5.25
Erkoc, 2024	Retrospective cohort	Turkey	122	390	84 (68.9%)†	284 (72.8%)†	45.56 ± 12.58†	46.94 ± 12.12†	25.27 ± 4.34†	25.44 ± 3.56†
Geavlete, 2024	Prospective cohort	Romania	35	35	10 (28.6%)	14 (40.0%)	NS	NS	NS	NS
Hongbo, 2025	Retrospective cohort	China	124	109	84 (67.7%)	79 (72.5%)	52 (39–64)*	48 (38–58)*	24.4 (21.9–27.1)*	25.3 (23.4–27.7)*
Hua, 2025	Retrospective cohort	China	81	101	54 (66.7%)	74 (73.3%)	49.48 ± 12.68	46.86 ± 12.90	24.98 ± 2.75	25.06 ± 3.68
Huang, 2023	Retrospective cohort	China	103	103	71 (68.9%)	68 (66.0%)	54.5 ± 11.0	54.7 ± 10.7	26.3 ± 4.2	26.5 ± 4.9
Liu, 2025	RCT	China	101	102	53 (52.5%)	58 (56.9%)	44.5 ± 6.1	43.2 ± 5.7	25.1 ± 2.9	24.7 ± 3.1
Mazzon, 2025	Prospective cohort	Multinational (UK, Italy, Singapore, Turkey, Egypt, Canada, Hong Kong, India, UAE)	184	184	105 (57.1%)	97 (52.7%)	56 (48–66)*	57 (50–67)*	26.4 (24.0–29.4)*	22.4 (20.7–24.1)*
Qian, 2022	Retrospective cohort	China	81	81	52 (64.2%)	56 (69.1%)	51.0 (42.0–57.0)*	50.0 (43.0–57.0)*	23.7 (22.1–26.1)*	24.2 (21.8–26.3)*
Rico, 2025	Retrospective cohort	Argentina	48	48	28 (58.3%)	29 (60.4%)	54 (41–67)*	57 (48–67)*	27.6 (23.3–30.4)*	25.8 (24–30)*
Tai, 2025	Retrospective cohort	Taiwan	36	34	25 (69.4%)	16 (47.1%)	57.5 (47–65)*	54.5 (47.5–65.8)*	26.3 ± 4.4	26.8 ± 4.3
Uslu, 2024	Prospective cohort	Turkey	43	45	29 (67.4%)	27 (60.0%)	56 (39–69)*	55 (42–61)*	27 (23–32)*	26 (22–29)*
Wang T, 2025	Retrospective cohort	China	62	134	39 (62.9%)	107 (79.9%)	56.87 ± 13.28	49.72 ± 13.82	24.57 ± 3.23	24.77 ± 3.09
Wang L, 2024	Retrospective cohort	China	71	74	47 (66.2%)	51 (68.9%)	45.87 ± 14.90	46.30 ± 13.99	28.50 ± 5.77	28.32 ± 5.12
Xu M, 2025	Prospective cohort	China	113	113	75 (66.4%)	80 (70.8%)	45.6 ± 11.8	46.2 ± 11.6	24.6 ± 2.8	24.1 ± 3.1
Xu XY, 2026	Retrospective cohort	China	53	58	31 (58.5%)	35 (60.3%)	46.09 ± 15.59	43.93 ± 13.27	22.68 ± 1.18	22.91 ± 1.23
Ying, 2024	Retrospective cohort	China	103	138	65 (63.1%)	103 (74.6%)	53.08 ± 12.99	53.92 ± 14.75	24.56 ± 3.37	24.91 ± 3.67
Yu, 2024	Retrospective cohort	China	152	152	75 (49.3%)	80 (52.6%)	51.1 ± 12.2	50.5 ± 11.8	24.3 ± 2.8	23.8 ± 2.8
Zhang, 2023	Retrospective cohort	China	102	112	55 (53.9%)	71 (63.4%)	47.69 ± 9.18	46.75 ± 11.87	24.25 ± 2.97	23.54 ± 3.37
Zhao, 2025	Retrospective cohort	China	69	83	43 (62.3%)	51 (61.4%)	45.7 ± 12.3	44.8 ± 12.6	NS	NS
Zhu, 2025	Retrospective cohort	China	36	33	20 (54.9%)	18 (55.0%)	46.58 ± 8.34	46.48 ± 11.40	25.64 ± 3.27	25.61 ± 3.42
Zhu, 2019	Retrospective cohort	China	165	165	109 (66.1%)	109 (66.1%)	53.9 ± 13.4	51.7 ± 15.8	22.9 ± 2.6	23.1 ± 3.4
Zhu, 2024	RCT	Multinational (China, Philippines, Malaysia, Turkey)	160	160	89 (55.6%)	96 (60.0%)	53.0 (45.0–64.0)*	52.0 (40.0–61.8)*	24.8 (22.4–26.5)*	24.2 (21.9–27.0)*

Values are mean ± standard deviation unless marked. *Median (interquartile range or range) as reported by the primary authors. †Whole cohort of Erkoc et al. 2024, which entered the meta-analysis as two strata by stone size (80 and 42 patients with S-UAS; 248 and 142 with T-UAS). BMI, body mass index; NS, not specified; RCT, randomized controlled trial; S-UAS, suction ureteral access sheath; T-UAS, traditional ureteral access sheath. Percentages use the number of patients in each arm; for Zhu et al. 2025 they are the inverse-probability-weighted values reported by the authors

**Table 2:** Perioperative outcomes and complications in the included studies

Study (author, year)	Operative time, min		Postoperative stay, days		Postoperative fever, events		Postoperative sepsis, events		Septic shock, events		Early SFR, *n* (%)		Late SFR, *n* (%)		Severe complications (grade III/IV or derived), events		Clavien-Dindo V, events	
	S-UAS	T-UAS	S-UAS	T-UAS	S-UAS	T-UAS	S-UAS	T-UAS	S-UAS	T-UAS	S-UAS	T-UAS	S-UAS	T-UAS	S-UAS	T-UAS	S-UAS	T-UAS
Alhefnawy, 2026	58 ± 11	58 ± 13	1 (1–2)*	1 (1–7)*	1 (1.4%)	5 (6.9%)	NS	NS	NS	NS	72 (98.6%)	59 (81.9%)	NS	NS	NS	NS	NS	NS
Anil, 2026	60.3 ± 25.7	71.9 ± 31.2	NS	NS	4 (4.4%)	12 (13.3%)	3 (3.3%)	5 (5.6%)	1 (1.1%)	2 (2.2%)	81 (90.0%)	71 (78.9%)	NS	NS	1 (1.1%)	2 (2.2%)	0 (0.0%)	0 (0.0%)
Arikan, 2025	50 (15–90)*	57.5 (25–90)*	NS	NS	4 (3.8%)	19 (18.6%)	NS	NS	NS	NS	50 (47.6%)	24 (23.5%)	79 (75.2%)	70 (68.6%)	0 (0.0%)	0 (0.0%)	0 (0.0%)	0 (0.0%)
Barbour, 2025	75 [63–97]*	67 [56–91]*	NS	NS	NS	NS	NS	NS	NS	NS	NS	NS	7 (50.0%)	7 (53.8%)	0 (0.0%)	0 (0.0%)	0 (0.0%)	0 (0.0%)
Cacciatore, 2025	55.25 [43.73–118.35]*	61.36 [46.73–125.35]*	2.76 [1.22–4.01]*	2.86 [1.75–3.97]*	1 (1.6%)	4 (5.7%)	0 (0.0%)	0 (0.0%)	0 (0.0%)	0 (0.0%)	NS	NS	59 (95.2%)	47 (67.1%)	0 (0.0%)	0 (0.0%)	0 (0.0%)	0 (0.0%)
Chen, 2024	101.17 ± 25.64	86.23 ± 20.35	1.53 ± 0.52	1.62 ± 0.63	1 (0.8%)	6 (5.3%)	NS	NS	0 (0.0%)	0 (0.0%)	109 (87.2%)	83 (73.5%)	119 (95.2%)	97 (85.8%)	0 (0.0%)	4 (3.5%)	0 (0.0%)	0 (0.0%)
Chiang, 2025	95.9 ± 49.8	98.4 ± 40.85	3 ± 3.69	2.79 ± 2.32	NS	NS	1 (2.0%)	2 (3.8%)	NS	NS	41 (80.4%)	32 (60.4%)	42 (82.4%)	40 (75.5%)	2 (3.9%)	2 (3.8%)	0 (0.0%)	0 (0.0%)
Erkoc, 2024 (< 2 cm)	40.48 ± 10.32	42.64 ± 11.68	1.72 ± 0.32	1.84 ± 0.28	4 (5.0%)	20 (8.1%)	2 (2.5%)	9 (3.6%)	NS	NS	NS	NS	72 (90.0%)	221 (89.1%)	2 (2.5%)	9 (3.6%)	0 (0.0%)	0 (0.0%)
Erkoc, 2024 (2–3 cm)	57.78 ± 11.72	65.64 ± 13.86	1.98 ± 0.46	2.02 ± 0.68	4 (9.5%)	18 (12.7%)	2 (4.8%)	10 (7.0%)	NS	NS	NS	NS	36 (85.7%)	110 (77.5%)	2 (4.8%)	10 (7.0%)	0 (0.0%)	0 (0.0%)
Geavlete, 2024	52.5	50.28	1.085	1	0 (0.0%)	2 (5.7%)	NS	NS	NS	NS	34 (97.1%)	29 (82.9%)	NS	NS	0 (0.0%)	0 (0.0%)	0 (0.0%)	0 (0.0%)
Hongbo, 2025	94.8 [60–139.2]*	79.8 [60–120]*	2 [2–3]*	3 [2–4]*	22 (17.7%)	32 (29.4%)	3 (2.4%)	11 (10.1%)	NS	NS	95 (76.6%)	46 (42.2%)	99 (79.8%)	62 (56.9%)	9 (7.3%)	24 (22.0%)	0 (0.0%)	0 (0.0%)
Hua, 2025	35.54 ± 15.35	37.63 ± 15.98	NS	NS	NS	NS	NS	NS	NS	NS	72 (88.9%)	91 (90.1%)	NS	NS	0 (0.0%)	0 (0.0%)	0 (0.0%)	0 (0.0%)
Huang, 2023	37.7 ± 20.1	40.3 ± 18.9	NS	NS	3 (2.9%)	4 (3.9%)	NS	NS	0 (0.0%)	0 (0.0%)	81 (78.6%)	52 (50.5%)	97 (94.2%)	78 (75.7%)	0 (0.0%)	0 (0.0%)	0 (0.0%)	0 (0.0%)
Liu, 2025	53.7 ± 4.9	54.8 ± 5.1	2.1 ± 0.7	2.2 ± 0.5	2 (2.0%)	9 (8.8%)	1 (1.0%)	8 (7.8%)	NS	NS	87 (86.1%)	72 (70.6%)	93 (92.1%)	84 (82.4%)	1 (1.0%)	8 (7.8%)	0 (0.0%)	0 (0.0%)
Mazzon, 2025	49.99 ± 23.56	50.02 ± 15.71	1 [0–2]*	2 [2–2]*	1 (0.5%)	0 (0.0%)	0 (0.0%)	0 (0.0%)	0 (0.0%)	0 (0.0%)	NS	NS	182 (98.9%)	164 (89.1%)	0 (0.0%)	0 (0.0%)	0 (0.0%)	0 (0.0%)
Qian, 2022	72.9 ± 28.1	80.0 ± 29.5	NS	NS	3 (3.7%)	12 (14.8%)	NS	NS	NS	NS	70 (86.4%)	58 (71.6%)	72 (88.9%)	67 (82.7%)	1 (1.2%)	10 (12.3%)	0 (0.0%)	0 (0.0%)
Rico, 2025	33.5 [26.3–44.5]*	57.5 [40–65]*	NS	NS	NS	NS	NS	NS	NS	NS	NS	NS	45 (93.8%)	36 (75.0%)	0 (0.0%)	0 (0.0%)	0 (0.0%)	0 (0.0%)
Tai, 2025	60 ± 26.5	69.2 ± 30.1	1.3 ± 0.8	1.4 ± 0.8	3 (8.3%)	2 (5.9%)	0 (0.0%)	0 (0.0%)	0 (0.0%)	0 (0.0%)	30 (83.3%)	26 (76.5%)	32 (88.9%)	30 (88.2%)	0 (0.0%)	2 (5.9%)	0 (0.0%)	0 (0.0%)
Uslu, 2024	55 [48–65]*	62 [59–72]*	2 [2–2]*	2 [2–2]*	1 (2.3%)	4 (8.9%)	0 (0.0%)	0 (0.0%)	0 (0.0%)	0 (0.0%)	NS	NS	35 (81.4%)	33 (73.3%)	0 (0.0%)	0 (0.0%)	0 (0.0%)	0 (0.0%)
Wang L, 2024	39.03 ± 18.01	49.73 ± 20.77	1.57 ± 0.82	2.30 ± 1.62	5 (7.0%)	14 (18.9%)	2 (2.8%)	8 (10.8%)	1 (1.4%)	2 (2.7%)	63 (88.7%)	56 (75.7%)	66 (93.0%)	63 (85.1%)	3 (4.2%)	4 (5.4%)	0 (0.0%)	0 (0.0%)
Wang T, 2025	55.23 ± 23.53	48.83 ± 22.91	3.15 ± 0.51	3.10 ± 0.45	1 (1.6%)	3 (2.2%)	0 (0.0%)	0 (0.0%)	0 (0.0%)	0 (0.0%)	54 (87.1%)	117 (87.3%)	NS	NS	0 (0.0%)	0 (0.0%)	0 (0.0%)	0 (0.0%)
Xu M, 2025	79.5 ± 6.8	65.2 ± 7.3	2.1 ± 1.1	2.2 ± 1.3	2 (1.8%)	6 (5.3%)	0 (0.0%)	4 (3.5%)	NS	NS	97 (85.8%)	82 (72.6%)	111 (98.2%)	102 (90.3%)	0 (0.0%)	4 (3.5%)	0 (0.0%)	0 (0.0%)
Xu XY, 2026	37.8 ± 6.07	46.59 ± 6.36	2.70 ± 0.70	2.88 ± 0.75	4 (7.5%)	5 (8.6%)	NS	NS	NS	NS	51 (96.2%)	47 (81.0%)	NS	NS	0 (0.0%)	0 (0.0%)	0 (0.0%)	0 (0.0%)
Ying, 2024	69.39 ± 21.69	65.93 ± 24.99	2.35 ± 0.50	2.41 ± 0.69	3 (2.9%)	7 (5.1%)	12 (11.7%)	18 (13.0%)	0 (0.0%)	1 (0.7%)	79 (76.7%)	88 (63.8%)	92 (89.3%)	102 (73.9%)	12 (11.7%)	18 (13.0%)	0 (0.0%)	0 (0.0%)
Yu, 2024	56.5 ± 13.9	59.9 ± 16.2	1.3 ± 0.68	1.4 ± 0.73	9 (5.9%)	28 (18.4%)	NS	NS	NS	NS	116 (76.3%)	11 (7.2%)	144 (94.7%)	142 (93.4%)	0 (0.0%)	0 (0.0%)	0 (0.0%)	0 (0.0%)
Zhang, 2023	55.25 ± 11.42	59.36 ± 15.59	2.86 ± 1.11	2.76 ± 1.51	4 (3.9%)	11 (9.8%)	5 (4.9%)	10 (8.9%)	1 (1.0%)	3 (2.7%)	88 (86.3%)	84 (75.0%)	93 (91.2%)	91 (81.3%)	2 (2.0%)	7 (6.3%)	0 (0.0%)	0 (0.0%)
Zhao, 2025	119.8 ± 10.4	93.2 ± 9.8	4.3 ± 0.7	4.4 ± 0.5	NS	NS	NS	NS	NS	NS	58 (84.1%)	57 (68.7%)	60 (87.0%)	70 (84.3%)	NS	NS	0 (0.0%)	0 (0.0%)
Zhu, 2019	49.7 ± 16.3	57.0 ± 14	1.1 ± 1.18	1.3 ± 1.15	9 (5.5%)	23 (13.9%)	4 (2.4%)	14 (8.5%)	1 (0.6%)	3 (1.8%)	136 (82.4%)	118 (71.5%)	143 (88.8%)	131 (82.9%)	2 (1.2%)	4 (2.4%)	0 (0.0%)	0 (0.0%)
Zhu, 2024	45.0 ± 23.2	40.9 ± 19.1	0.93 ± 0.825	0.95 ± 0.77	9 (5.6%)	28 (17.5%)	NS	NS	0 (0.0%)	1 (0.6%)	130 (81.3%)	79 (49.4%)	140 (87.5%)	112 (70.0%)	0 (0.0%)	1 (0.6%)	0 (0.0%)	0 (0.0%)
Zhu, 2025	63.02 ± 8.17	73.92 ± 15.56	3.15 ± 0.46	5.16 ± 1.35	0 (0.0%)	4 (12.5%)	0 (0.0%)	0 (0.0%)	0 (0.0%)	0 (0.0%)	NS	NS	27 (75.5%)	16 (48.9%)	0 (0.0%)	0 (0.0%)	0 (0.0%)	0 (0.0%)

Values are mean ± standard deviation, median (range) or median [interquartile range] as reported by the primary authors; NS, not specified; SFR, stone-free rate; S-UAS, suction ureteral access sheath; T-UAS, traditional ureteral access sheath. *Median (range) or median [interquartile range]. Early SFR: assessment up to 3 weeks after surgery; late SFR: assessment at 4 weeks or later; percentages use the number of patients assessed at each time point or, when it was not reported (Chiang 2025), all treated patients. For Zhu 2025 all percentages are the inverse-probability-weighted values reported by the authors. Geavlete 2024 reported operative time as means with ranges (30–75 and 30–90 min) and hospital stay as means without dispersion. Severe complications: Clavien-Dindo grade III or IV events in the studies that graded each event, and, in the others, events requiring endoscopic or surgical reintervention or intensive care together with sepsis, systemic inflammatory response syndrome or septic shock as described by the authors; the meta-analysis of severe complications added grade V events to this column

The suction sheath had a flexible tip in 23 studies, a fixed tip in 5 [48, 52, 59, 64, 72], and devices of both designs were pooled without description of the tip in 1 [53]. The negative pressure applied, reported in 18 studies in several units, ranged from approximately 15 to 450 mmHg (Supplementary Table 2). Sheath calibre, reported for both arms in 25 studies, ranged from 9.5/11.5 to 13/15 Fr across the studies, most often 10/12 to 12/14 Fr. The two arms used the same calibre or the same distribution of calibres in 19 of these 25 studies; the comparator was one size smaller in one [52], and calibre differed between arms in five [51, 58, 61, 63, 67]. Pre-stenting was reported per arm in 15 studies and ranged from 6% to 76%; it differed between arms by 10% points or more in four studies, with more pre-stented patients in the S-UAS arm in three (58% versus 18% [63]; 67% versus 57% [58]; 24% versus 13%, stent or nephrostomy [51]) and in the T-UAS arm in one (66% versus 76% [48]), and by less than 10 points in the other 11; two studies excluded pre-stented patients [46, 62], and 12 did not report it per arm. No study was restricted to first-time stone formers. Recurrent stone formers were reported in three studies (25% to 64%) [48, 58, 64], previous stone surgery in four [45, 57, 69, 72], one study excluded patients with previous stone surgery [52], and the remaining 21 gave no per-arm information on stone history.

Seven studies had no stone-free assessment before 4 weeks, because the first imaging was scheduled at 4 weeks to 3 months [48, 49, 52, 58, 60, 62, 71], and six had no assessment at 4 weeks or later, because follow-up ended at 2 to 4 weeks [45, 46, 53, 55, 63, 66]; the study that assessed at 2 to 4 weeks was placed in the early group [55]. Clavien-Dindo grades were reported per event in 10 studies. In the others, severe complications were derived from the events described; in two of them no event type could be mapped to the definition, because one reported infection, fever and hematuria without grades or reinterventions [45] and the other reported only a total [70].

### Early stone-free rate

**Fig. 2:**
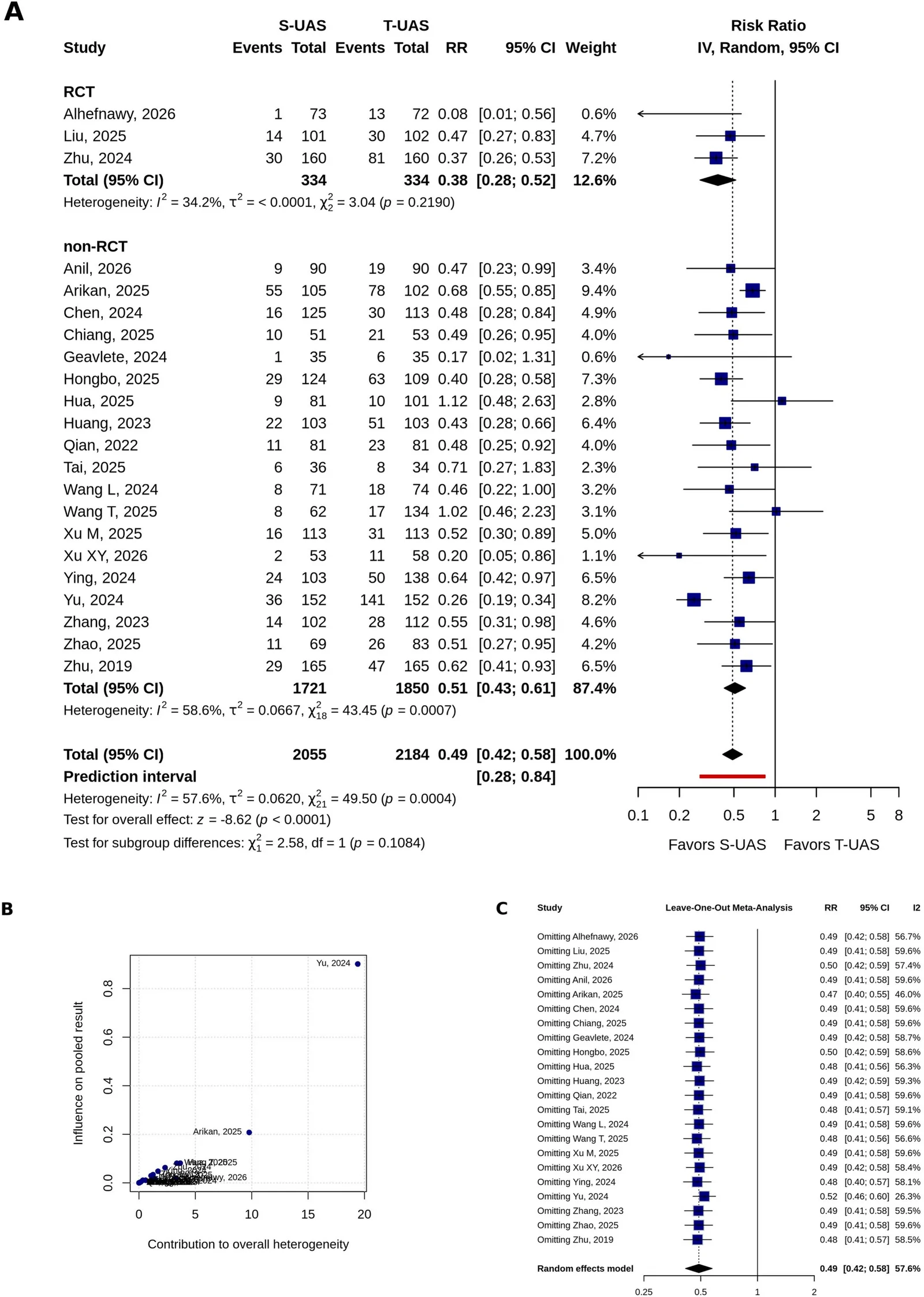
Early residual fragments (assessment up to 3 weeks after surgery). (A) Forest plot; (B) Baujat plot; (C) leave-one-out sensitivity analysis

Data from 22 studies [45–47, 50, 51, 53–57, 59, 61, 63–70, 72, 73] and 4,239 patients (2,055 S-UAS versus 2,184 T-UAS) were pooled. S-UAS reduced the risk of residual fragments (RR 0.49; 95% CI 0.42 to 0.58; *p* < 0.001), with moderate heterogeneity (I² 57.6%, 95% CI 32 to 74%; τ² 0.062; Q 49.5, *p* < 0.001) and a prediction interval of 0.28 to 0.84 (Fig. 2A). The Baujat plot pointed to Yu et al. [68] and Arikan et al. [47] as the main contributors to heterogeneity (Fig. 2B). Omitting Yu et al. lowered I² to 26.3% (RR 0.52; 95% CI 0.46 to 0.60) and omitting Arikan et al. to 46.0% (RR 0.47; 95% CI 0.40 to 0.55), and the direction and significance of the effect were preserved in all 22 iterations (Fig. 2C). The three RCTs favored S-UAS (RR 0.38; 95% CI 0.28 to 0.52; I² 34.2%), as did the 19 non-RCTs (RR 0.51; 95% CI 0.43 to 0.61; I² 58.6%), with no difference between designs (*p* = 0.108).

### Late stone-free rate

**Fig. 3:**
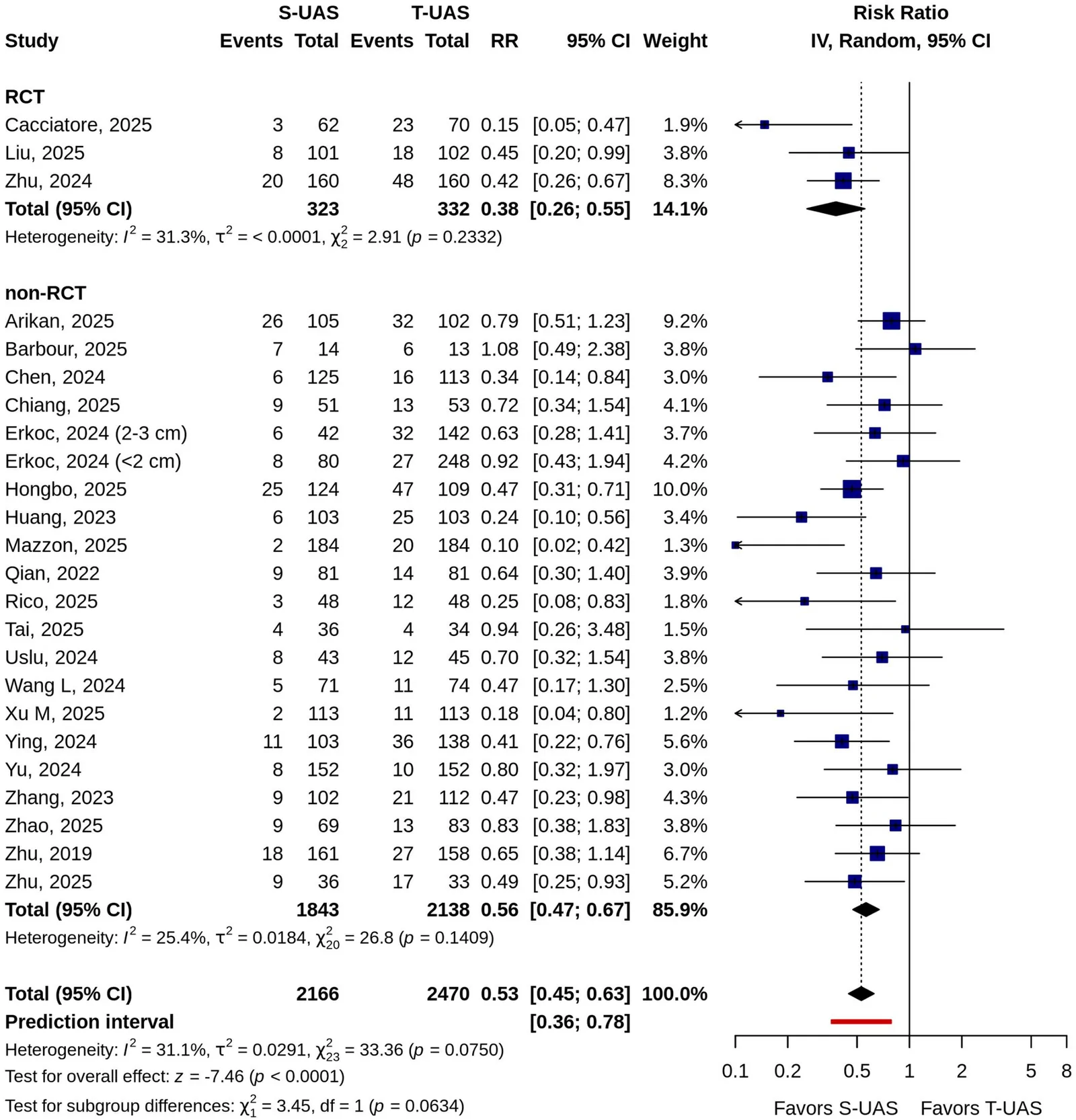
Forest plot for late residual fragments (assessment at 4 weeks or later)

Twenty-three studies [47–52, 54, 56–62, 64, 65, 67–73] with 4,636 patients (2,166 versus 2,470) reported stone-free status at 4 weeks or later. S-UAS again reduced residual fragments (RR 0.53; 95% CI 0.45 to 0.63; *p* < 0.001), with low heterogeneity (I² 31.1%, 95% CI 0 to 58%; τ² 0.029) and a prediction interval of 0.36 to 0.78 (Fig. 3). The effect was present in the three RCTs (RR 0.38; 95% CI 0.26 to 0.55; I² 31.3%) and in the non-RCTs (RR 0.56; 95% CI 0.47 to 0.67; I² 25.4%), and the difference between designs did not reach significance (*p* = 0.063). In the 16 studies that assessed both time points, the crude proportion of patients with residual fragments fell from 42.9% to 20.5% with T-UAS and from 19.9% to 10.6% with S-UAS between the early and the late assessment, so the absolute difference narrowed from 23 to 10% points while the risk ratio remained at 0.48 (95% CI 0.41 to 0.57) and 0.53 (95% CI 0.44 to 0.63).

### Postoperative fever

Twenty-four studies [45–47, 49, 50, 52–54, 56–59, 61–69, 71–73] with 4,970 patients (2,301 versus 2,669) reported postoperative fever, with 100 events in the S-UAS arms and 278 in the T-UAS arms. S-UAS reduced the risk of fever (RR 0.43; 95% CI 0.34 to 0.53; *p* < 0.001), with no detectable heterogeneity (I² 0%, 95% CI 0 to 44%) and a prediction interval of 0.34 to 0.54 (Supplementary Fig. 1). The estimate was similar in the four RCTs (RR 0.29; 95% CI 0.16 to 0.53) and in the 20 non-RCTs (RR 0.46; 95% CI 0.36 to 0.58).

### Postoperative sepsis

Sixteen studies [46, 49, 51, 52, 54, 57, 58, 61–65, 67, 69, 71, 72] with 3,311 patients (1,465 versus 1,846) reported sepsis, with 35 events in the S-UAS arms and 99 in the T-UAS arms; six comparisons had no event in either arm. S-UAS reduced the risk of sepsis (RR 0.47; 95% CI 0.31 to 0.72; *p* < 0.001; I² 0%), but the prediction interval reached unity (0.22 to 1.01) (Supplementary Fig. 2). The Mantel-Haenszel model gave RR 0.50 (95% CI 0.34 to 0.73). The effect was present in the non-RCTs (RR 0.50; 95% CI 0.33 to 0.77), and only one RCT was informative, favoring S-UAS (RR 0.13; 95% CI 0.02 to 0.99) [57].

### Septic shock

Fourteen studies [46, 49, 50, 56, 58, 61–64, 67, 69, 71–73] with 2,797 patients (1,342 versus 1,455) reported septic shock, which occurred in 4 patients treated with S-UAS (0.30%) and 12 treated with T-UAS (0.82%), all in six comparisons. The point estimate favored S-UAS but the confidence interval included unity (RR 0.41; 95% CI 0.15 to 1.15; *p* = 0.090; I² 0%; prediction interval 0.11 to 1.58), with the same result under Mantel-Haenszel weighting (Supplementary Fig. 3). The non-RCT subgroup gave the same picture (RR 0.42; 95% CI 0.14 to 1.25), and the single informative RCT had one event [73].

### Severe postoperative complications

Twenty-seven studies (28 comparisons) with 5,307 patients (2,458 versus 2,849) contributed to this outcome, with 37 events in the S-UAS arms and 109 in the T-UAS arms; 13 comparisons had no event in either arm. S-UAS reduced the risk of severe complications (RR 0.48; 95% CI 0.33 to 0.69; *p* < 0.001; I² 0%), and the prediction interval did not cross unity (0.32 to 0.72) (Supplementary Fig. 4). The effect was significant in the non-RCTs (RR 0.50; 95% CI 0.35 to 0.73) and in the RCTs, where it rested on 10 events (RR 0.17; 95% CI 0.03 to 0.95; 1 versus 9 events). When the analysis was restricted to the 10 studies that reported Clavien-Dindo grades per event, the point estimate was similar and the interval widened (RR 0.46; 95% CI 0.21 to 1.01; 8 versus 20 events), and when the studies whose count derived in whole or in part from sepsis or SIRS were removed, the result was RR 0.42 (95% CI 0.19 to 0.89; 8 versus 24 events).

### Operative time

All 29 studies (5,604 patients) reported operative time. There was no difference between S-UAS and T-UAS (MD − 1.24 min; 95% CI − 4.74 to 2.26; *p* = 0.49), with very high heterogeneity (I² 96.0%; τ² 83.6; Q 719.2) and a prediction interval of − 20.3 to 17.8 min (Supplementary Fig. 5A). The Baujat plot identified Zhao et al. [70], Xu M et al. [65], Xu XY et al. [66], Chen et al. [50] and Rico et al. [60] as the main contributors (Supplementary Fig. 5B), and no single omission reduced I² below 93.9% (Supplementary Fig. 5C). Neither the RCTs (MD 0.19 min; 95% CI − 2.44 to 2.82; I² 36.5%) nor the non-RCTs (MD − 1.44 min; 95% CI − 5.42 to 2.54; I² 96.5%) showed a difference. Six studies had entered operative time from medians; without them the difference was MD − 0.54 min (95% CI − 4.34 to 3.25).

### Length of hospital stay

Twenty-two studies reported LOS, two of them without a usable measure of dispersion [53, 62], so 20 studies (21 comparisons) with 4,313 patients (1,964 versus 2,349) were pooled. The stay was shorter with S-UAS (MD − 0.32 days; 95% CI − 0.53 to − 0.10; *p* = 0.004), but heterogeneity was very high (I² 90.2%; τ² 0.22) and the prediction interval crossed zero (− 1.33 to 0.70 days) (Supplementary Fig. 6A). The non-RCTs gave MD − 0.30 days (95% CI − 0.54 to − 0.06; I² 89.3%) and the four RCTs MD − 0.39 days (95% CI − 0.98 to 0.19; I² 94.4%; p for subgroup difference 0.77); the RCT estimate depended on one trial in which the stay in the comparator arm ranged up to 7 days [45], while the other three trials found differences of 0.2 day or less; the estimated mean for that comparator arm is sensitive to its skewed range, and without this trial the pooled difference was − 0.27 days. The Baujat plot identified Zhu et al. [71], Alhefnawy et al. [45], Mazzon et al. [58], the smaller-stone stratum of Erkoc et al. [52] and Wang T et al. [63] as the main contributors (Supplementary Fig. 6B), and the leave-one-out estimates ranged from − 0.34 to − 0.24 days (Supplementary Fig. 6C). Four studies had entered LOS from medians; without them the difference was MD − 0.20 days (95% CI − 0.40 to 0.00; *p* = 0.052).

### Risk of bias

Among the four RCTs, one was at low risk of bias [73] and three raised some concerns [45, 49, 57] (Supplementary Fig. 7A). Among the 25 non-RCTs, three were at low risk [56, 59, 64], 18 at moderate risk, and four at serious risk, in all cases because of confounding [53, 58, 63, 71] (Supplementary Fig. 7B). Studies at low risk of bias carried 5% to 21% of the weight in the non-randomized pooled estimates, and the low-risk trial carried 58% to 69% of the RCT weight for stone clearance and fever but 0% to 29% for sepsis, severe complications and operative time.

### Subgroup analysis by tip design

For early residual fragments, the estimate was RR 0.49 (95% CI 0.40 to 0.58) in the 18 studies with tip-flexible sheaths and RR 0.55 (95% CI 0.40 to 0.76) in the three with fixed-tip sheaths (*p* = 0.49). For late residual fragments, the benefit was larger with tip-flexible sheaths (RR 0.47; 95% CI 0.39 to 0.58; 18 studies) than with fixed-tip sheaths (RR 0.72; 95% CI 0.53 to 0.97; 5 studies; *p* = 0.026) (Supplementary Figs. 8 and 9). Under the classification by brand, which moves the three studies naming the ClearPetra sheath to the fixed-tip group, the difference was present at both time points (early RR 0.46 versus 0.63, *p* = 0.017; late RR 0.44 versus 0.74, *p* = 0.001). The two designs did not differ for fever (RR 0.42 versus 0.44; *p* = 0.86), sepsis (RR 0.49 versus 0.40; *p* = 0.67), septic shock (RR 0.41 versus 0.41), severe complications (RR 0.45 versus 0.53; *p* = 0.73), operative time (MD − 0.4 versus − 5.5 min; *p* = 0.081) or LOS (MD − 0.33 versus − 0.21 days; *p* = 0.49) (Supplementary Table 3). Restricting the binary outcomes to tip-flexible sheaths gave estimates within 0.06 of the main ones.

### Small-study effects and sensitivity analyses

By Harbord’s and Peters’ tests, funnel plots were symmetric for early residual fragments (Harbord *p* = 0.49; Peters *p* = 0.89), late residual fragments (*p* = 0.75; *p* = 0.40), fever (*p* = 0.79; *p* = 0.85), sepsis (*p* = 0.13; *p* = 0.85) and severe complications (*p* = 0.19; *p* = 0.67), and by Egger’s test for operative time (*p* = 0.37) and LOS (*p* = 0.065) (Supplementary Table 4; Supplementary Fig. 10); Egger’s test, which is not designed for sparse binary data, was significant for sepsis (*p* = 0.036) and for no other outcome. Trim-and-fill imputed one to two studies for stone clearance and fever without changing any estimate by more than 0.01, three for sepsis (adjusted RR 0.57; 95% CI 0.38 to 0.85) and four for severe complications (adjusted RR 0.56; 95% CI 0.37 to 0.83). Septic shock had too few informative studies for these tests. Re-including the two studies with a suction comparator, excluding the six studies with a minority of patients outside the eligibility criteria, or excluding the four studies at serious risk of bias changed the estimates for the binary outcomes by at most 0.02, with two exceptions on the continuous outcomes: without the six studies outside the criteria, operative time was shorter with S-UAS (MD − 3.94 min; 95% CI − 6.81 to − 1.07), and without the studies at serious risk of bias the difference in LOS fell to − 0.21 days (95% CI − 0.36 to − 0.06) (Supplementary Table 5).

### Certainty of evidence

The certainty of evidence was moderate for early and late residual fragments in the RCTs, downgraded for imprecision, and for late residual fragments in the non-RCTs, downgraded for risk of bias; it was low for early residual fragments in the non-RCTs, downgraded for risk of bias and inconsistency. For fever it was moderate in both designs. For sepsis it was very low in the RCTs and low in the non-RCTs, for septic shock low and very low, for severe complications very low in both designs, because of risk of bias, imprecision and the indirectness of the operational definition, and for operative time low in the RCTs and very low in the non-RCTs, where inconsistency was very serious (Supplementary Table 6).

## Discussion

In the present update, 29 studies and 5,604 patients were pooled, against 8 studies in our 2025 review [17], and the conclusions of that review held: S-UAS halved the risk of residual fragments at early and late assessment, reduced fever and complications, and did not change operative time. The other syntheses published since 2025 have reached the same direction of effect. Xie et al. [10] pooled 10 comparative studies and 2,105 patients and found odds ratios of 2.87 for immediate and 2.98 for 1-month stone-free status, with less fever (OR 0.31) and no difference in operative time; Mittal et al. [20] pooled 15 studies and 3,030 patients with a trial sequential analysis that judged the evidence for immediate stone-free status sufficient; Uy et al. [19] pooled 34 comparative and non-comparative studies and 7,193 patients and found higher stone-free rates and fewer infections with FANS; and Li et al. [18] restricted the synthesis to six randomized trials of suction-assisted RIRS, including suction catheters, and reported an OR of 4.00 for stone-free status. In the present study, the comparative evidence base is the largest assembled so far, the two designs of suction sheath were analyzed separately, and the certainty of evidence was rated for each outcome.

The late assessment is the one with clinical meaning, and the comparison of the two time points needs care. In the 16 studies that assessed both, the crude proportion of patients with residual fragments halved between the early and the late assessment in both arms, from 43% to 21% with T-UAS and from 20% to 11% with S-UAS, as fragments passed spontaneously; the absolute difference therefore narrowed from 23 to 10% points, although the risk ratio stayed close to 0.5. The T-UAS arms did catch up in part. What remained at 1 to 3 months was an absolute difference of about one patient in ten, and these are the fragments that grow, cause colic and lead to reintervention: Chiang et al. [51] reported auxiliary procedures in 5.9% of patients treated with a suction sheath and 22.6% of those treated with a traditional sheath, and Xu et al. [66] in 2 versus 10 patients. Fragments removed by suction during lithotripsy no longer depend on spontaneous passage, whereas a traditional sheath relies on basket retrieval and passive drainage and leaves a larger residual burden [76–78].

Heterogeneity at the early assessment was largely attributable to two studies. In Yu et al. [68], the stone-free rate of 7.2% in the T-UAS arm reflects a zero-fragment definition on computed tomography and basket extraction in 100% of the comparator patients, in a single-surgeon series with one sheath model. Arikan et al. [47] found an early benefit that had disappeared by 1 month, with a different device and pressure range than most other studies. Omitting either study reduced I² without changing the estimate, which points to differences in stone-free definitions and comparator technique rather than to inconsistency in the effect of suction. The late assessment, where these differences matter less, had low heterogeneity.

The subgroup analysis by tip design was post hoc and compares studies rather than patients, and its result should be read as a hypothesis. The late benefit was larger with the flexible design (RR 0.47 versus 0.72), and the difference grew when the ClearPetra sheath was classified as fixed-tip by brand. A tip that reaches the calyx aspirates fragments where they lie, whereas a fixed tip parked at the pyeloureteral junction depends on fragments being carried to it, so the direction is plausible. Against it stand three facts. The fixed-tip studies are older, two of them historical-control comparisons, and all five relied wholly or partly on radiography for the stone-free definition, so era and imaging are partly confounded with design. The classification relied on the authors’ descriptions, and the same brand name was described as a bendable sheath by some authors and not described by others. Finally, the direct evidence points the other way: Uy et al. [19] found no difference in stone-free rate between FANS and fixed-tip suction sheaths in the studies that compared the two head to head, although the two such reports that we read in full both favored the flexible tip for stone clearance [74, 75]. Both designs reduced fever and sepsis; for severe complications the fixed-tip estimate had a wide interval that included unity (RR 0.53; 95% CI 0.26 to 1.07), and no claim of equivalence is made.

The reduction in infectious complications followed a gradient of severity: fever (RR 0.43) and severe complications (RR 0.48) with prediction intervals below unity, sepsis (RR 0.47) with a prediction interval reaching unity, and septic shock (RR 0.41) with a confidence interval that included unity. Part of this gradient is arithmetic, because in the studies without Clavien-Dindo grading the same sepsis and SIRS events enter both the sepsis and the severe-complication counts; the estimate without those studies (RR 0.42) shows that the finding does not depend on them. The estimates for sepsis and septic shock do not conflict. The point estimate for septic shock is as low as that for sepsis, but it rests on 16 events in 2,797 patients, and a confidence interval of 0.15 to 1.15 is compatible with an 85% reduction as well as with no effect; the absence of significance reflects the number of events, not a different direction. A trial powered for septic shock at the rates observed here (0.30% versus 0.82%; two-sided α 0.05, 80% power, 1:1 allocation) would need about 6,300 patients. In addition, sepsis was defined by Sepsis-3 criteria in one study, by qSOFA or SIRS criteria in others, and not at all in most [79], and SIRS-based definitions include patients with transient fever, so the sepsis outcome is broader and more frequent than septic shock. The mechanism behind the gradient is probably the control of intrarenal pressure: suction through the sheath keeps the renal pelvic pressure below the threshold for pyelovenous and pyelolymphatic backflow of irrigation fluid, bacteria and endotoxin, while maintaining a clear field [59, 80–82]. The comparisons are not clean, however, because several studies used pump irrigation in the suction arm and manual irrigation in the control arm [54, 67, 73], and irrigation method alone changes intrarenal pressure. Tzelves et al. [83] reviewed the newer sheaths that add real-time pressure sensing and automated feedback to keep the pressure within a preset range and reported series with near-zero sepsis; whether pressure monitoring adds anything to suction alone remains to be tested.

The efficiency outcomes were inconsistent, and the sources of inconsistency are identifiable. Operative time varied with the lithotripsy technique (dusting under continuous aspiration versus fragmentation and basketing, with baskets used in 100% of the comparator patients in some series [68, 73]), with the laser platform and settings (a 30 W laser in the suction arm against 100 W in the comparator in one study [61], and thulium fiber laser in 84% versus 4% in another [58]), with the suction protocol (pressures from 15 to 450 mmHg), with surgeon experience, since Barbour et al. [48] attributed their longer suction procedures to the learning curve, and with case selection, since two studies assigned larger or more complex stones to the suction sheath [53, 63]. Under these conditions a pooled mean difference of 1 min has no clinical meaning, and the prediction interval of − 20 to 18 min is the honest summary. Hospital stay depends on discharge policy more than on the device: three of the four RCTs found differences of 0.2 day or less, the difference lost significance without the four studies that entered medians (MD − 0.20 days; 95% CI − 0.40 to 0.00) and shrank to − 0.21 days without the studies at serious risk of bias, the largest differences came from a propensity-weighted series with two days [71], three observational series with two thirds of a day to a full day [54, 58, 64] and one trial whose comparator arm had stays of up to 7 days [45], and one series discharged both arms on the day of surgery [55]. The shorter stay with S-UAS therefore describes the included cohorts rather than an effect of the sheath.

Most of the evidence is observational, and confounding is the main threat. Only three of the 25 non-randomized studies were at low risk of bias, and these contributed 5% to 21% of the weight to the pooled estimates. However, the four RCTs gave the same direction of effect for every binary outcome, with larger effects for stone clearance (RR 0.38) than the non-RCTs, and removing the four studies at serious risk of bias changed the binary estimates by at most 0.02. By Harbord’s and Peters’ tests, small-study effects were not detected for any outcome, and Egger’s test was significant only for sepsis; trim-and-fill moved the estimates for sepsis and severe complications toward unity (RR 0.57 and 0.56), which with 11 to 15 informative studies is better read as a reminder of low power than as evidence of missing studies.

The limitations of the present study are the following. First, stone-free status was defined with thresholds from zero to 4 mm and assessed with computed tomography in all patients in only 11 of the 23 studies with late assessment, so part of the between-study variation reflects imaging rather than the sheath. Second, complication grading was not uniform: only 10 studies reported Clavien-Dindo grades per event, and in the others severe complications were derived from the events described, which is why the outcome is called operational and rated very low certainty. Third, sheath calibre, pre-stenting and stone history were reported unevenly, calibre and pre-stenting were unbalanced in a few studies, and their influence could not be examined. Fourth, sepsis and septic shock were defined differently or not defined, co-interventions such as irrigation method, basketing and laser platform differed between arms in several studies, and six studies included a minority of patients outside the eligibility criteria. Fifth, the classification of tip design relied on the descriptions of the primary authors, and the subgroup analysis was post hoc. Finally, S-UAS cost more than traditional sheaths; the two single-center cost analyses published so far found a lower cost per stone-free patient [46] and savings from fewer baskets [48], but a formal cost-effectiveness analysis is still lacking. However, with respect to the first point, the effect was the same at the early and the late assessment, and with respect to the fourth, the sensitivity analyses on eligibility and risk of bias changed no binary estimate by more than 0.02, and those restricted to graded studies or to counts not derived from infectious events kept the direction of the effect on severe complications (RR 0.46 and 0.42). A randomized trial comparing tip-flexible and fixed-tip suction sheaths, with computed tomography at 3 months, sepsis defined by Sepsis-3 criteria and cost recorded per patient, would answer the questions this review leaves open.

## Conclusion

In this updated systematic review and meta-analysis of 29 studies and 5,604 patients, suction ureteral access sheaths were associated with half the risk of residual fragments at early and late assessment and with fewer episodes of fever, sepsis and severe complications than traditional sheaths, without a difference in operative time. The certainty of evidence is moderate for stone clearance and fever, except for early clearance in non-randomized studies, and low or very low for the other outcomes; the effect on septic shock is imprecise, and the larger late benefit of the tip-flexible design is a hypothesis that needs a randomized trial.

## Supplementary Information

Below is the link to the electronic supplementary material.

Supplementary Material 1

## Data Availability

No datasets were generated or analysed during the current study.

## Declarations

### Use of artificial intelligence

A large language model (Claude, Anthropic) was used to revise the manuscript text, to write and check the analysis code, and to pre-extract the device characteristics, sheath calibres, pre-stenting, stone history and timing of stone-free assessment from the primary reports with verbatim supporting quotations, which the authors then checked against the reports. The analyses of the submitted version had been carried out independently by the authors in R and were reproduced exactly before the revised analyses were run. All eligibility decisions, all outcome data extraction, all analytical choices and all interpretation are the authors’ own. The authors reviewed every output and take full responsibility for the content of this article.
