# Supplementary material for: Suction versus traditional ureteral access sheaths in retrograde intrarenal surgery: an updated systematic review and meta-analysis of stone-free rates and complications
Source: World J Urol. 2026 Nov 5;44(1):789. doi: 10.1007/s00345-026-06873-5 (PMC13739964; doi:10.1007/s00345-026-06873-5)
Supplement: Supplementary file 1 [file 345_2026_6873_MOESM1_ESM.docx]

**Supplementary Material**

Suction versus traditional ureteral access sheaths in retrograde intrarenal surgery: an updated systematic review and meta-analysis of stone-free rates and complications

Supplementary tables and supplementary figures are numbered in independent series (Supplementary Table 1, 2, …; Supplementary Figure 1, 2, …) and are cited by their full names in the main text.

**Supplementary Table 1. Complete search strategy for each database (inception to March 2026).**

| **Database** | **Search strategy** |
| --- | --- |
| MEDLINE (PubMed) | (Perfusion OR suction* OR suctioning OR aspirat* OR vacuum OR "negative pressure" OR "pressure control" OR evacu* OR tip-bendable OR "tip flexible" OR "flexible tip" OR tip-flexible) AND (("ureteral access sheath"[tiab]) OR ("ureteral sheath"[tiab]) OR ("ureteral access"[tiab]) OR ("suctioning ureteral access sheath"[tiab]) OR (UAS[tiab]) OR (FANS[tiab]) OR (S-UAS[tiab]) OR ("negative pressure"[tiab] AND "ureteral access sheath"[tiab]) OR (vacuum[tiab] AND "ureteral access sheath"[tiab]) OR ((fluid[tiab] OR irrigation[tiab]) AND (aspirat*[tiab] OR suction*[tiab]) AND "ureteral access sheath"[tiab])) AND (("retrograde intrarenal surgery"[tiab]) OR (RIRS[tiab]) OR ("flexible ureteroscopy"[tiab]) OR ("flexible ureteroscope"[tiab]) OR ("digital flexible ureteroscopy"[tiab]) OR (ureterorenoscopy[tiab]) OR ("ureteroscopic lithotripsy"[tiab]) OR ("ureteral stones"[tiab]) OR ("kidney stones"[tiab]) OR (urolithiasis[tiab]) OR ("renal calculi"[tiab])) |
| Embase | (Perfusion OR suction* OR suctioning OR aspirat* OR vacuum OR 'negative pressure' OR 'pressure control' OR evacu* OR tip-bendable OR 'tip flexible' OR 'flexible tip' OR tip-flexible) AND (('ureteral access sheath':ti,ab) OR ('ureteral sheath':ti,ab) OR ('ureteral access':ti,ab) OR ('suctioning ureteral access sheath':ti,ab) OR (UAS:ti,ab) OR (FANS:ti,ab) OR (S-UAS:ti,ab) OR ('negative pressure':ti,ab AND 'ureteral access sheath':ti,ab) OR (vacuum:ti,ab AND 'ureteral access sheath':ti,ab) OR ((fluid:ti,ab OR irrigation:ti,ab) AND (aspirat*:ti,ab OR suction*:ti,ab) AND 'ureteral access sheath':ti,ab)) AND (('retrograde intrarenal surgery':ti,ab) OR (RIRS:ti,ab) OR ('flexible ureteroscopy':ti,ab) OR ('flexible ureteroscope':ti,ab) OR ('digital flexible ureteroscopy':ti,ab) OR (ureterorenoscopy:ti,ab) OR ('ureteroscopic lithotripsy':ti,ab) OR ('ureteral stones':ti,ab) OR ('kidney stones':ti,ab) OR (urolithiasis:ti,ab) OR ('renal calculi':ti,ab)) |
| Scopus | (Perfusion OR suction* OR suctioning OR aspirat* OR vacuum OR "negative pressure" OR "pressure control" OR evacu* OR tip-bendable OR "tip flexible" OR "flexible tip" OR tip-flexible) AND (("ureteral access sheath") OR ("ureteral sheath") OR ("ureteral access") OR ("suctioning ureteral access sheath") OR (UAS) OR (FANS) OR (S-UAS) OR ("negative pressure" AND "ureteral access sheath") OR (vacuum AND "ureteral access sheath") OR ((fluid OR irrigation) AND (aspirat* OR suction*) AND "ureteral access sheath")) AND (("retrograde intrarenal surgery") OR (RIRS) OR ("flexible ureteroscopy") OR ("flexible ureteroscope") OR ("digital flexible ureteroscopy") OR (ureterorenoscopy) OR ("ureteroscopic lithotripsy") OR ("ureteral stones") OR ("kidney stones") OR (urolithiasis) OR ("renal calculi")) |
| Web of Science | (Perfusion OR suction* OR suctioning OR aspirat* OR vacuum OR "negative pressure" OR "pressure control" OR evacu* OR tip-bendable OR "tip flexible" OR "flexible tip" OR tip-flexible) AND (("ureteral access sheath") OR ("ureteral sheath") OR ("ureteral access") OR ("suctioning ureteral access sheath") OR (UAS) OR (FANS) OR (S-UAS) OR ("negative pressure" AND "ureteral access sheath") OR (vacuum AND "ureteral access sheath") OR ((fluid OR irrigation) AND (aspirat* OR suction*) AND "ureteral access sheath")) AND (("retrograde intrarenal surgery") OR (RIRS) OR ("flexible ureteroscopy") OR ("flexible ureteroscope") OR ("digital flexible ureteroscopy") OR (ureterorenoscopy) OR ("ureteroscopic lithotripsy") OR ("ureteral stones") OR ("kidney stones") OR (urolithiasis) OR ("renal calculi")) |
| Cochrane Library | (Perfusion OR suction* OR suctioning OR aspirat* OR vacuum OR "negative pressure" OR "pressure control" OR evacu* OR tip-bendable OR "tip flexible" OR "flexible tip" OR tip-flexible) AND (("ureteral access sheath":ti,ab) OR ("ureteral sheath":ti,ab) OR ("ureteral access":ti,ab) OR ("suctioning ureteral access sheath":ti,ab) OR (UAS:ti,ab) OR (FANS:ti,ab) OR (S-UAS:ti,ab) OR ("negative pressure":ti,ab AND "ureteral access sheath":ti,ab) OR (vacuum:ti,ab AND "ureteral access sheath":ti,ab) OR ((fluid:ti,ab OR irrigation:ti,ab) AND (aspirat*:ti,ab OR suction*:ti,ab) AND "ureteral access sheath":ti,ab)) AND (("retrograde intrarenal surgery":ti,ab) OR (RIRS:ti,ab) OR ("flexible ureteroscopy":ti,ab) OR ("flexible ureteroscope":ti,ab) OR ("digital flexible ureteroscopy":ti,ab) OR (ureterorenoscopy:ti,ab) OR ("ureteroscopic lithotripsy":ti,ab) OR ("ureteral stones":ti,ab) OR ("kidney stones":ti,ab) OR (urolithiasis:ti,ab) OR ("renal calculi":ti,ab)) |

**Supplementary Table 2. Device, sheath calibre, pre-stenting, stone history, stone size, timing of stone-free assessment and complication grading in the included studies.**

| **Study** | **Design** | **Country** | **Suction sheath (brand)** | **Tip design** | **Negative pressure** | **Calibre S-UAS / T-UAS (Fr)** | **Comparator sheath** | **Stone size, S-UAS vs T-UAS** | **Pre-stented, S-UAS vs T-UAS** | **Stone history** | **Early assessment (≤3 wk)** | **Late assessment (≥4 wk)** | **Clavien-Dindo grades per event** | **Notes** |
| --- | --- | --- | --- | --- | --- | --- | --- | --- | --- | --- | --- | --- | --- | --- |
| Alhefnawy, 2026 | RCT (three-arm) | Egypt | Elephant Type II FANS (maker NR) | Flexible | 2–7 kPa | 11/13 / 11/13 | Boston Scientific | 22 ± 2 vs 23 ± 2 mm (14–27 mm) | 5.5% vs 6.9% | Previous renal surgery 26% vs 43% | 15 d, NCCT, <4 mm | Not assessed | No | Three-arm RCT; PCN arm not used. Fever not defined. Severe complications not derivable (no grades or reinterventions) |
| Anil, 2026 | Retrospective, PSM | Turkey | ClearPetra (Well Lead); flexible distal tip described | Flexible* | NR | 10/12 81%, 11/13 14%, 12/14 4% / 10/12 83%, 11/13 14%, 12/14 2% | NR | 18.2 ± 6.9 vs 17.8 ± 6.7 mm | Pre-stented excluded | NR | 3 wk, LD-NCCT, ≤4 mm | Not assessed | Yes | Urosepsis reported as grade 2 (3 vs 5) and septic shock as grade 4 (1 vs 2); severe complications = grade IV only |
| Arikan, 2025 | Retrospective, multicenter | Turkey | ClearPetra 10/12 (Well Lead); bendable tip described | Flexible* | 50–150 mmHg | 10/12 / 10/12 | Plasti-med | 13 (7–20) vs 12 (6–20) mm; <2 cm | NR | NR | 1 wk, LD-CT, zero fragments | 1 mo, LD-CT, zero fragments | No | Arms from different hospitals. Sepsis and septic shock not reported |
| Barbour, 2025 | Prospective vs historical control | United Kingdom | ClearPetra | Fixed | NR | NR / NR | NR | 10 (8–12) vs 10 (7–12) mm; partial staghorn 3 vs 1 | 66% vs 76% | Prior stone history 64% vs 60% | Not assessed | <3 mo, CT/KUB/US, >4 mm (imaged 14 vs 13) | No | Historical control. Late imaging in 14 vs 13 patients, at clinicians' discretion |
| Cacciatore, 2025 | RCT (method not described) | Italy | ClearPetra (Well Lead) or NP-UAS (Innovex) | Flexible* | Adjustable, NR | 10/12 / 10/12 | Rocamed Bi-Flex | 13.5 (10–24) vs 13.5 (11–20) mm | NR | NR | Not assessed | 1 mo, LD-NCCT, >3 mm | Yes | Randomization method not described. Laser platform differed by arm (pulsed thulium vs holmium) |
| Chen, 2024 | Retrospective | China | TFS-UAS (maker NR) | Flexible | Wall suction, NR | 12/14 / 12/14 | NR | 2.8 ± 1.6 vs 2.7 ± 1.4 cm (2–4 cm) | NR | NR | POD1, KUB + CT, <2 mm | 30 d, CT, <2 mm | No (steinstrasse needing surgery counted) | Steinstrasse requiring surgery (0 vs 4) counted as severe. Fever >38 °C |
| Chiang, 2025 | Retrospective | Taiwan | Bendable S-UAS (maker NR) | Flexible | 50–100 mmHg | 10/12 59%, 11/13 33%, 12/14 6% / 11/13 | NR | 13.7 ± 6.5 vs 14.3 ± 7.0 mm (≤30 mm) | DJ 9.8% + PCN 13.7% vs DJ 7.5% + PCN 5.7% | NR | ≤24 h, KUB, >4 mm | ≈1 mo, KUB, >4 mm | No | Bilateral cases and staghorn stones included; different ureteroscope calibres by arm. Urosepsis (1 vs 2) and ureteral stricture (1 vs 0) counted as severe complications |
| Erkoc, 2024 | Retrospective (two strata) | Turkey | ClearPetra | Fixed | NR | 11/13 / 10/12 | Plastimed | 19.8 ± 8.2 vs 18.5 ± 7.8 mm (≤3 cm) | 17.5% vs 12% (<2 cm); 16.6% vs 11.2% (2–3 cm) | Prior stone surgery excluded; SWL history 35% vs 32% | Not assessed | 3 mo, KUB/NCCT, <4 mm | No | Two strata (<2 cm, 2–3 cm) entered separately. Sepsis counts (2/9, 2/10) used for severe complications; fever and sepsis not defined |
| Geavlete, 2024 | Prospective, three-arm | Romania | ClearPetra or YiGao Elephant II | Mixed | 85–150 mmHg | 10/12 / 10/12 | NR | 19.1 vs 18.3 mm (≤25 mm) | NR | NR | 3 wk, US/KUB, >3 mm | Not assessed | Yes (0 vs 0) | Three-arm; comparator = group 1 (conventional sheath, no in-scope suction). Suction arm also used direct in-scope suction |
| Hongbo, 2025 | Retrospective, historical control | China | Tip-bendable S-UAS (Yi Gao) | Flexible | 0.02–0.04 MPa | 11/13–12/14 / 11/13–12/14 | Wei Li | 1.4 (1.2–1.8) vs 1.5 (1.2–1.7) cm | NR | NR | Immediate (timing NS), KUB/NCCT, ≤2 mm | 30 d, KUB/NCCT, ≤2 mm | No | Historical control; pump irrigation in suction arm only. Sepsis = qSOFA ≥2 (3 vs 11); SIRS (9 vs 24) used for severe complications; early assessment timing not specified |
| Hua, 2025 | Retrospective | China | Navigable suction sheath (Copper) | Flexible | NR | 11/13 / 11/13 | Boston Scientific | 13.3 ± 5.0 vs 14.5 ± 7.5 mm | 10% overall (per arm NR) | NR | 2–4 wk, NCCT, >4 mm | Not assessed | Yes (0 vs 0) | Same-day surgery; suction implied by device name only. About half of stones ureteral |
| Huang, 2023 | Retrospective, PSM | China | FV-UAS (Copper) | Flexible | 100–300 cmH2O | 11/13 61%, 12/14 39% / 11/13 62%, 12/14 38% | Copper | 1.7 ± 0.5 vs 1.7 ± 0.6 cm (<3 cm) | 28% vs 25% | NR | POD1, CT, <3 mm | 30 d, CT, <3 mm | No | Fever within 72 h, threshold not given. Late assessment on day 30 treated as 4 weeks |
| Liu, 2025 | RCT | China | FV-UAS (Wellead) | Flexible | 85–90 cmH2O | 10/12 / NR | NR | 1.6 ± 0.4 vs 1.5 ± 0.5 cm (1–2 cm) | NR | Prior stone surgery 17% vs 15% | POD1, ULD-CT, ≤2 mm | 4 wk, modality NS | No | Per-protocol analysis (24 of 227 excluded after randomization). Fever ≥38.5 °C; urosepsis undefined (1 vs 8), used for severe complications |
| Mazzon, 2025 | Prospective multicenter, PSM | International | FANS (maker NR) | Flexible | NR | 10/12 41%, 11/13 37%, 12/14 21% / 10/12 61%, 11/13 14%, 12/14 25% | NR | volume 1.40 vs 1.40 cm³ | 67% vs 57% | Recurrent formers 25% vs 37% | Not assessed | 30 d, NCCT, >4 mm or multiple fragments | Yes | Arms from different centers; thulium fiber laser 84% vs 4%. Fever = fever requiring antibiotics; sepsis = Sepsis-3. 30-day CT treated as 4 weeks |
| Qian, 2022 | Retrospective, PSM | China | Suctioning UAS (maker NR) | Fixed | 0.01 MPa | 12/14 / 12/14 | Cook | 19 (17–23) vs 20 (17–23) mm | 16% vs 20% | NR | POD1, KUB/US, <4 mm | 1 mo, KUB/NCCT, <4 mm | No | Fever >38 °C within 48 h; SIRS (1 vs 10) used for severe complications; sepsis not reported |
| Rico, 2025 | Retrospective, historical control | Argentina | FANS (maker NR) | Flexible | NR | 10/12 / 10/12 | NR | volume 895 vs 805 mm³ | 23% vs 25% | NR | Not assessed | 4 wk, NCCT, zero fragments | No (grade ≤2 only) | Historical control; only Clavien ≤2 events reported |
| Tai, 2025 | Retrospective, alternating allocation | Taiwan | ClearPetra or Reborn CS | Flexible* | 50 cmHg (as reported) | 11/13 (10/12 if <1 cm) / 11/13 (12/14 if larger) | Rocamed Bi-flex evo | 14.5 ± 4.8 vs 13.6 ± 5.7 mm | 25% vs 21% | NR | 24 h and 7 d, KUB + US, >2 mm | 30 d, KUB + US, >2 mm | Yes | Alternating allocation; 30 W laser in suction arm vs 100 W. Residual >2 mm used as the stone-free threshold |
| Uslu, 2024 | Prospective, non-randomized | Turkey | ClearPetra (Well Lead); tip-bendable per authors | Flexible* | NR | NR / 9.5/11.5 | Cook | 10 (8–17) vs 11 (8–13) mm (<20 mm) | Pre-stented excluded | NR | Not assessed | 1 mo, NCCT, <3 mm | No (events itemized) | Complications itemized as fever, transient hematuria, colic due to clots and subcapsular hematoma; none required intervention |
| Wang L, 2024 | Retrospective | China | ClearPetra (Well Lead) | Fixed | 150–200 mmHg | 12/14 / 12/14 | Cook | 14.7 ± 4.2 vs 14.5 ± 4.0 mm (<25 mm) | NR | Recurrent formers 35% vs 32% | POD1, KUB, <4 mm | 1 mo, KUB/NCCT, <4 mm | Yes | Fever >38 °C within 48 h; sepsis = qSOFA ≥2. Severe complications = perforation (grade III) and septic shock (grade IV): 3 vs 4 |
| Wang T, 2025 | Retrospective | China | TFS-UAS (Copper) | Flexible | 150–200 mmHg | 11/13 51%, 12/14 49% / 10/12 10%, 11/13 83%, 12/14 7% (≥15 mm subgroup) | Boston Scientific | 17.7 ± 7.8 vs 12.6 ± 5.1 mm (≤40 mm) | 58% vs 18% | First-time formers 69% (overall) | Intraoperative and 2 wk, CT, ≥3 mm | Not assessed | No | Overall-cohort outcomes taken from the authors' supplementary table; pre-stenting 58% vs 18%; stones up to 40 mm included |
| Xu M, 2025 | Prospective, non-randomized | China | FV-UAS (Wellead) | Flexible | 100 cmH2O | 12/14 / 12/14 | Wellead | 2.2 ± 0.5 vs 2.1 ± 0.8 cm (<3 cm) | 27% vs 31% | NR | POD1, KUB, ≤2 mm | 1 mo, KUB/US/CT, ≤2 mm | No | Urosepsis undefined (0 vs 4), used for severe complications |
| Xu XY, 2026 | Retrospective | China | FANS (HuaMei YWF) | Flexible | NR | 10/12 / 10/12 | Pulin | 13.8 ± 1.5 vs 13.5 ± 1.3 mm (1–2 cm) | NR | NR | 1 d and 2 wk, KUB, >2 mm | Not assessed | No | Fever not defined; no severe complications reported |
| Ying, 2024 | Retrospective | China | TFS-UAS (Zhejiang Yigao) | Flexible | 100–160 cmH2O | 10 F ID 34%, 12.5 F ID 66% / 11/13 24%, 12/14 40%, 13/15 36% | Boston Scientific | 1.55 ± 0.58 vs 1.56 ± 0.64 cm | 25% vs 20% | NR | POD1, KUB (CT in 17%), <2 mm | 3 mo, KUB/US/CT, <2 mm | No | Pump irrigation in suction arm only. SIRS (12 vs 18) used for sepsis and for severe complications; one septic shock |
| Yu, 2024 | Retrospective, matched, historical control | China | Flexible UAS (Zhangjiagang) | Flexible | 2–7 kPa | 12/14 / 12/14 | Zhangjiagang | 15.5 ± 2.0 vs 15.2 ± 1.9 mm (1–2 cm) | 7% vs 9% | NR | POD1, NCCT, zero fragments | 1 mo, NCCT, zero fragments | No | Historical control; basket in 100% of comparator patients. Fever >38 °C |
| Zhang, 2023 | Retrospective | China | NTFS-UAS (Zhangjiagang Huamei) | Flexible | 20–60 kPa | 12/14 / 12/14 | KYB | 18.5 ± 4.7 vs 18.2 ± 4.5 mm | 45% vs 42% | Prior ipsilateral surgery 20% vs 26% | POD1, KUB (CT in 36%), <2 mm | 30 d, KUB/CT, <2 mm | Yes | Fever >38 °C; urosepsis (grade II) plus septic shock counted as sepsis; severe = grades III–IV |
| Zhao, 2025 | Retrospective | China | Bendable suction sheath (Zhejiang Yigao) | Flexible | 150–200 mmHg | 10/12 / 10/12 | NR | 1.4 ± 0.5 vs 1.3 ± 0.5 cm (<2 cm) | NR | NR | 1 wk, CT, >4 mm | 1 mo, CT, >4 mm | No (totals only) | About 40% ureteral stones; bilateral cases included. Complications reported as totals only |
| Zhu, 2019 | Retrospective, matched, historical control | China | Suctioning UAS (KYB) | Fixed | 3–8 kPa | 12/14 / 12/14 | KYB | 18.2 ± 5.2 vs 17.4 ± 4.7 mm | NR | Prior ipsilateral surgery 33% vs 36% | POD1, KUB, <2 mm | 1 mo, KUB/NCCT, <2 mm (143/161 vs 131/158) | Yes | Historical control. Late denominators exclude 4 and 7 patients lost to follow-up. Urosepsis plus septic shock counted as sepsis; severe = grades III–IV |
| Zhu, 2024 | RCT, multicenter | International | Tip-bendable S-UAS (maker NR) | Flexible | 80–120 mmHg | 10/12 18%, 11/13 64%, 12/14 18% / 10/12 13%, 11/13 70%, 12/14 16% | NR | 14 (10–20) vs 11 (9–16) mm (≤3 cm) | 12% vs 14% | NR | ≤24 h, endoscopy + KUB + US, <2 mm | 3 mo, LD-CT, ≤2 mm | Yes | Pump irrigation and no basketing in suction arm; basket in all comparator patients. Fever >38.5 °C; septic shock = Sepsis-3; about 35% proximal ureteral stones; bilateral (8%) and staghorn (4%) cases included |
| Zhu, 2025 | Retrospective, IPTW | China | FANS (maker NR) | Flexible | NR | 10/11 / NR | NR | 1.64 ± 0.24 cm in both arms (1–2 cm) | NR | NR | Not assessed | 30 d, CT, <4 mm | No | Counts back-calculated from IPTW-weighted percentages; allocation by surgeon preference |

S-UAS, suction ureteral access sheath; T-UAS, traditional ureteral access sheath; FANS, flexible and navigable suction ureteral access sheath; PSM, propensity score matching; IPTW, inverse probability of treatment weighting; NR, not reported; NS, not specified; NCCT, non-contrast computed tomography; LD, low dose; ULD, ultra-low dose; KUB, kidney-ureter-bladder radiography; US, ultrasonography; POD1, first postoperative day; DJ, double-J stent; PCN, percutaneous nephrostomy. Stone size is the largest diameter (mean ± SD, median (range) or median [IQR]) per arm as reported. Tip design was classified from the description given by the primary authors (see Methods). *The ClearPetra sheath, whose original model has a fixed tip, is named alone (Anil, Arikan, Uslu) or pooled with a tip-flexible device (Cacciatore, Tai) while the authors describe a flexible or bendable tip; these studies move to the fixed-tip or mixed group in the classification by brand. Two reports were excluded after full-text re-reading because the comparator sheath was also connected to suction: Wu et al. 2025 (comparator described as a traditional negative-pressure sheath, Well Lead 12 Fr, used with a perfusion and suction system) and Xiong et al. 2025 (comparator connected to a negative-pressure device at 50–100 mmHg).

**Supplementary Table 3. Subgroup analysis by tip design of the suction sheath and sensitivity analysis restricted to tip-flexible sheaths.**

| **Outcome** | **Classification by description: tip-flexible, k; estimate (95% CI)** | **Classification by description: fixed-tip, k; estimate (95% CI)** | **p, flexible vs fixed (description)** | **Classification by brand: tip-flexible, k; estimate (95% CI)** | **Classification by brand: fixed-tip, k; estimate (95% CI)** | **p, flexible vs fixed (brand)** | **Tip-flexible only (description): estimate (95% CI); prediction interval** |
| --- | --- | --- | --- | --- | --- | --- | --- |
| Early residual fragments | 18; RR 0.49 (0.40 to 0.58); I² 64.0% | 3; RR 0.55 (0.40 to 0.76); I² 0% | 0.49 | 15; RR 0.46 (0.38 to 0.56) | 5; RR 0.63 (0.53 to 0.75) | 0.017 | RR 0.49 (0.40 to 0.58); 0.27 to 0.89 |
| Late residual fragments | 18; RR 0.47 (0.39 to 0.58); I² 34.9% | 6 comparisons (5 studies); RR 0.72 (0.53 to 0.97); I² 0% | 0.026 | 14; RR 0.44 (0.37 to 0.54) | 8 comparisons (7 studies); RR 0.74 (0.58 to 0.94) | 0.001 | RR 0.47 (0.39 to 0.58); 0.29 to 0.77 |
| Postoperative fever | 19; RR 0.42 (0.33 to 0.55); I² 0% | 5 comparisons (4 studies); RR 0.44 (0.29 to 0.68); I² 0% | 0.86 | 14; RR 0.45 (0.34 to 0.59) | 8 comparisons (7 studies); RR 0.38 (0.27 to 0.56) | 0.52 | RR 0.42 (0.33 to 0.55); 0.32 to 0.56 |
| Postoperative sepsis | 7 informative; RR 0.49 (0.27 to 0.87); I² 11.2% | 4 informative; RR 0.40 (0.20 to 0.79); I² 0% | 0.67 | 6 informative; RR 0.44 (0.22 to 0.89) | 5 informative; RR 0.43 (0.24 to 0.80) | 0.96 | RR 0.49 (0.27 to 0.87); 0.15 to 1.53 |
| Septic shock | 4 informative; RR 0.41 (0.11 to 1.54) | 2 informative; RR 0.41 (0.08 to 2.11) | 1.00 | 3 informative; RR 0.38 (0.08 to 1.85) | 3 informative; RR 0.44 (0.11 to 1.69) | 0.89 | RR 0.41 (0.11 to 1.54); 0.05 to 3.52 |
| Severe complications | 10 informative; RR 0.45 (0.29 to 0.71); I² 5.2% | 5 informative; RR 0.53 (0.26 to 1.07); I² 0% | 0.73 | 8 informative; RR 0.43 (0.24 to 0.76) | 6 informative; RR 0.52 (0.26 to 1.04) | 0.65 | RR 0.45 (0.29 to 0.71); 0.23 to 0.88 |
| Operative time (min) | 23; MD −0.42 (−4.76 to 3.92); I² 96.6% | 6 comparisons (5 studies); MD −5.46 (−9.09 to −1.83); I² 69.6% | 0.081 | 18; MD 1.47 (−3.54 to 6.47) | 9 comparisons (8 studies); MD −6.34 (−8.74 to −3.94) | 0.006 | — |
| Length of hospital stay (days) | 17; MD −0.33 (−0.60 to −0.07); I² 91.7% | 4 comparisons (3 studies); MD −0.21 (−0.44 to 0.01); I² 67.2% | 0.49 | 15; MD −0.36 (−0.65 to −0.06) | 4 comparisons (3 studies); MD −0.21 (−0.44 to 0.01) | 0.44 | — |

Post hoc analysis. k, number of studies or comparisons contributing to the subgroup estimate; for sepsis, septic shock and severe complications, only comparisons with at least one event contribute. Classification by description follows the primary authors' description of the tip (Supplementary Table 2); the single study that pooled devices of both designs without describing the tip (Geavlete 2024) is excluded from the two-group test. Classification by brand moves Anil 2026, Arikan 2025 and Uslu 2024 (ClearPetra) to the fixed-tip group and Cacciatore 2025 and Tai 2025 (ClearPetra pooled with a tip-flexible device) to a mixed group excluded from the test. p values are for the difference between the flexible and fixed groups (Q test). Random-effects models with REML estimation of τ² (DerSimonian-Laird where REML did not converge).

**Supplementary Table 4. Assessment of small-study effects.**

| **Outcome** | **Studies (informative)** | **Harbord test, t (p)** | **Peters test, t (p)** | **Egger test, t (p)** | **Trim-and-fill: imputed studies; adjusted estimate (95% CI)** |
| --- | --- | --- | --- | --- | --- |
| Early residual fragments | 22 (22) | 0.71 (0.49) | −0.14 (0.89) | −0.43 (0.67) | 1; RR 0.49 (0.42 to 0.58) |
| Late residual fragments | 24 comparisons (24) | −0.32 (0.75) | 0.86 (0.40) | −1.56 (0.13) | 1; RR 0.54 (0.45 to 0.64) |
| Postoperative fever | 25 comparisons (25) | −0.27 (0.79) | −0.19 (0.85) | −0.79 (0.44) | 2; RR 0.44 (0.35 to 0.54) |
| Postoperative sepsis | 17 comparisons (11) | −1.67 (0.13) | −0.19 (0.85) | −2.46 (0.036) | 3; RR 0.57 (0.38 to 0.85) |
| Septic shock | 14 (6) | not assessed | not assessed | not assessed | not assessed |
| Severe complications | 28 comparisons (15) | −1.37 (0.19) | −0.43 (0.67) | −1.89 (0.082) | 4; RR 0.56 (0.37 to 0.83) |
| Operative time | 30 comparisons (30) | — | — | −0.90 (0.37) | — |
| Length of hospital stay | 21 comparisons (21) | — | — | −1.96 (0.065) | — |

Tests were applied to outcomes with at least ten informative studies (studies with at least one event in one arm). Harbord's and Peters' tests were used for the binary outcomes and Egger's test for the continuous outcomes; Egger's test is shown for the binary outcomes for completeness only, because it is unreliable for sparse binary data. Trim-and-fill was run on the random-effects model. Contour-enhanced funnel plots are shown in Supplementary Figure 10.

**Supplementary Table 5. Sensitivity analyses.**

| **Outcome** | **Analysis** | **Studies** | **Estimate (95% CI)** | **I²** |
| --- | --- | --- | --- | --- |
| Early residual fragments | Main analysis | 22 | RR 0.49 (0.42 to 0.58) | 57.6% |
|  | Including Wu 2025 and Xiong 2025 | 23 | RR 0.48 (0.41 to 0.56) | 58.4% |
|  | Excluding studies with patients outside criteria 4–6 | 17 | RR 0.49 (0.40 to 0.59) | 63.4% |
|  | Excluding studies at serious risk of bias | 20 | RR 0.48 (0.41 to 0.57) | 57.9% |
| Late residual fragments | Main analysis | 23 (24 comparisons) | RR 0.53 (0.45 to 0.63) | 31.1% |
|  | Including Wu 2025 and Xiong 2025 | 25 (26 comparisons) | RR 0.53 (0.45 to 0.62) | 26.4% |
|  | Excluding studies with patients outside criteria 4–6 | 18 (19 comparisons) | RR 0.51 (0.42 to 0.62) | 31.7% |
|  | Excluding studies at serious risk of bias | 21 (22 comparisons) | RR 0.54 (0.46 to 0.65) | 25.0% |
| Postoperative fever | Main analysis | 24 (25 comparisons) | RR 0.43 (0.34 to 0.53) | 0% |
|  | Including Wu 2025 and Xiong 2025 | 26 (27 comparisons) | RR 0.43 (0.35 to 0.53) | 0% |
|  | Excluding studies with patients outside criteria 4–6 | 21 (22 comparisons) | RR 0.44 (0.35 to 0.56) | 0% |
|  | Excluding studies at serious risk of bias | 20 (21 comparisons) | RR 0.43 (0.34 to 0.53) | 0% |
| Postoperative sepsis | Main analysis (inverse variance) | 16 (17 comparisons; 11 informative) | RR 0.47 (0.31 to 0.72) | 0% |
|  | Mantel-Haenszel model | 16 (17 comparisons; 11 informative) | RR 0.50 (0.34 to 0.73) | 0% |
|  | Excluding studies with patients outside criteria 4–6 | 10 informative | RR 0.47 (0.30 to 0.73) | 0% |
|  | Excluding studies at serious risk of bias | 11 informative | RR 0.47 (0.31 to 0.72) | 0% |
| Septic shock | Main analysis (inverse variance) | 14 (6 informative) | RR 0.41 (0.15 to 1.15) | 0% |
|  | Mantel-Haenszel model | 14 (6 informative) | RR 0.41 (0.15 to 1.15) | 0% |
|  | Including Wu 2025 and Xiong 2025 | 15 (7 informative) | RR 0.41 (0.15 to 1.08) | 0% |
|  | Excluding studies with patients outside criteria 4–6 | 5 informative | RR 0.42 (0.14 to 1.25) | 0% |
| Severe complications | Main analysis (operational definition) | 27 (28 comparisons; 15 informative) | RR 0.48 (0.33 to 0.69) | 0% |
|  | Including Wu 2025 and Xiong 2025 | 29 (30 comparisons; 16 informative) | RR 0.48 (0.33 to 0.69) | 0% |
|  | Restricted to studies reporting Clavien-Dindo grades per event | 10 (6 informative) | RR 0.46 (0.21 to 1.01) | 0% |
|  | Excluding studies whose count derived from sepsis or SIRS | 20 (7 informative) | RR 0.42 (0.19 to 0.89) | 0% |
|  | Excluding studies with patients outside criteria 4–6 | 12 informative | RR 0.48 (0.33 to 0.70) | 0% |
|  | Excluding studies at serious risk of bias | 15 informative | RR 0.48 (0.33 to 0.69) | 0% |
| Operative time | Main analysis | 29 (30 comparisons) | MD −1.24 min (−4.74 to 2.26) | 96.0% |
|  | Excluding studies with patients outside criteria 4–6 | 23 (24 comparisons) | MD −3.94 min (−6.81 to −1.07) | 94.5% |
|  | Excluding studies at serious risk of bias | 25 (26 comparisons) | MD −1.35 min (−5.28 to 2.59) | 96.4% |
|  | Excluding studies entered from medians | 23 (24 comparisons) | MD −0.54 min (−4.34 to 3.25) | 96.5% |
| Length of hospital stay | Main analysis | 20 (21 comparisons) | MD −0.32 d (−0.53 to −0.10) | 90.2% |
|  | Excluding studies with patients outside criteria 4–6 | 15 (16 comparisons) | MD −0.41 d (−0.68 to −0.14) | 92.0% |
|  | Excluding studies at serious risk of bias | 17 (18 comparisons) | MD −0.21 d (−0.36 to −0.06) | 79.0% |
|  | Excluding studies entered from medians | 16 (17 comparisons) | MD −0.20 d (−0.40 to 0.00) | 79.4% |

Wu 2025 and Xiong 2025 compared a tip-flexible suction sheath with a comparator sheath that was also connected to suction. Studies with a minority of patients outside eligibility criteria 4 to 6 (stones larger than 3 cm, staghorn stones or bilateral procedures): Barbour 2025, Chen 2024, Chiang 2025, Wang T 2025, Zhao 2025 and Zhu 2024. Studies at serious risk of bias (ROBINS-I): Geavlete 2024, Mazzon 2025, Wang T 2025 and Zhu 2025. Studies reporting Clavien-Dindo grades per event: Anil 2026, Cacciatore 2025, Geavlete 2024, Hua 2025, Mazzon 2025, Tai 2025, Wang L 2024, Zhang 2023, Zhu 2019 and Zhu 2024. Studies whose severe-complication count derived in whole or in part from sepsis or SIRS: Chiang 2025, Erkoc 2024, Hongbo 2025, Liu 2025, Qian 2022, Xu M 2025 and Ying 2024. Studies entered from medians (means and standard deviations estimated with the method of Wan et al. 2014): hospital stay, Alhefnawy 2026, Cacciatore 2025, Hongbo 2025 and Mazzon 2025; operative time, Arikan 2025, Barbour 2025, Cacciatore 2025, Hongbo 2025, Rico 2025 and Uslu 2024. Geavlete 2024 reported operative time as means with ranges (standard deviations estimated from the ranges). Geavlete 2024 (means without dispersion) and Uslu 2024 (interquartile range of zero in both arms) could not be pooled for hospital stay.

**Supplementary Table 6. GRADE summary of findings: suction versus traditional ureteral access sheath in retrograde intrarenal surgery.**

| **Outcome** | **№ studies** | **Design** | **Risk of bias** | **Inconsistency** | **Indirectness** | **Imprecision** | **Other** | **S-UAS** | **T-UAS** | **Relative effect (95% CI)** | **Absolute effect (95% CI)** | **Certainty** |
| --- | --- | --- | --- | --- | --- | --- | --- | --- | --- | --- | --- | --- |
| Early residual fragments (≤3 wk) | 3 | RCT | not serious | not serious | not serious | serious^c^ | none | 45/334 (13.5%) | 124/334 (37.1%) | RR 0.38 (0.28 to 0.52) | 229 fewer per 1,000 (266 fewer to 180 fewer) | ⨁⨁⨁◯ Moderate |
|  | 19 | non-RCT | serious^a^ | serious^b^ | not serious | not serious | none | 316/1,721 (18.4%) | 678/1,850 (36.6%) | RR 0.51 (0.43 to 0.61) | 180 fewer per 1,000 (211 fewer to 144 fewer) | ⨁⨁◯◯ Low |
| Late residual fragments (≥4 wk) | 3 | RCT | not serious | not serious | not serious | serious^c^ | none | 31/323 (9.6%) | 89/332 (26.8%) | RR 0.38 (0.26 to 0.55) | 167 fewer per 1,000 (199 fewer to 119 fewer) | ⨁⨁⨁◯ Moderate |
|  | 20 (21 comparisons) | non-RCT | serious^a^ | not serious | not serious | not serious | none | 190/1,843 (10.3%) | 406/2,138 (19.0%) | RR 0.56 (0.47 to 0.67) | 83 fewer per 1,000 (100 fewer to 63 fewer) | ⨁⨁⨁◯ Moderate |
| Postoperative fever | 4 | RCT | not serious | not serious | not serious | serious^c^ | none | 13/396 (3.3%) | 46/404 (11.4%) | RR 0.29 (0.16 to 0.53) | 81 fewer per 1,000 (96 fewer to 54 fewer) | ⨁⨁⨁◯ Moderate |
|  | 20 (21 comparisons) | non-RCT | serious^a^ | not serious | not serious | not serious | none | 87/1,905 (4.6%) | 232/2,265 (10.2%) | RR 0.46 (0.36 to 0.58) | 56 fewer per 1,000 (66 fewer to 43 fewer) | ⨁⨁⨁◯ Moderate |
| Postoperative sepsis | 2 (1 informative) | RCT | serious^a^ | not applicable | not serious | very serious^d^ | none | 1/163 (0.6%) | 8/172 (4.7%) | RR 0.13 (0.02 to 0.99) | 41 fewer per 1,000 (46 fewer to 0 fewer) | ⨁◯◯◯ Very low |
|  | 14 (15 comparisons; 10 informative) | non-RCT | serious^a^ | not serious | not serious | serious^c^ | none^e^ | 34/1,302 (2.6%) | 91/1,674 (5.4%) | RR 0.50 (0.33 to 0.77) | 27 fewer per 1,000 (36 fewer to 13 fewer) | ⨁⨁◯◯ Low |
| Septic shock | 2 (1 informative) | RCT | not serious | not applicable | not serious | very serious^d^ | none | 0/222 (0%) | 1/230 (0.4%) | RR 0.33 (0.01 to 8.12) | 3 fewer per 1,000 (4 fewer to 31 more) | ⨁⨁◯◯ Low |
|  | 12 (5 informative) | non-RCT | serious^a^ | not serious | not serious | very serious^d^ | none | 4/1,120 (0.4%) | 11/1,225 (0.9%) | RR 0.42 (0.14 to 1.25) | 5 fewer per 1,000 (8 fewer to 2 more) | ⨁◯◯◯ Very low |
| Severe complications (operational definition) | 3 (2 informative) | RCT | serious^a^ | not serious | serious^f^ | very serious^d^ | none | 1/323 (0.3%) | 9/332 (2.7%) | RR 0.17 (0.03 to 0.95) | 23 fewer per 1,000 (26 fewer to 1 fewer) | ⨁◯◯◯ Very low |
|  | 24 (25 comparisons; 13 informative) | non-RCT | serious^a^ | not serious | serious^f^ | serious^c^ | none^e^ | 36/2,135 (1.7%) | 100/2,517 (4.0%) | RR 0.50 (0.35 to 0.73) | 20 fewer per 1,000 (26 fewer to 11 fewer) | ⨁◯◯◯ Very low |
| Operative time (min) | 4 | RCT | serious^a^ | not serious | not serious | serious^c^ | none | 396 | 404 | — | MD 0.19 higher (2.44 lower to 2.82 higher) | ⨁⨁◯◯ Low |
|  | 25 (26 comparisons) | non-RCT | serious^a^ | very serious^b^ | not serious | serious^c^ | none | 2,204 | 2,600 | — | MD 1.44 lower (5.42 lower to 2.54 higher) | ⨁◯◯◯ Very low |

CI, confidence interval; RR, risk ratio; MD, mean difference; RCT, randomized controlled trial. Evidence from non-randomized studies assessed with ROBINS-I starts at high certainty. Absolute effects were calculated from the pooled control-group risk and the unrounded pooled RR. One set of rules was applied to both designs (see Methods).

a. Downgraded for risk of bias: studies at low overall risk of bias contributed less than half of the weight (5% to 21% of the non-randomized weight; the low-risk trial contributed 0% of the RCT weight for sepsis, 29% for severe complications and 28% for operative time).

b. Downgraded for inconsistency: I² of 58.6% for early residual fragments in non-randomized studies; I² of 96.5% for operative time in the non-randomized studies, with point estimates on both sides of the null (very serious).

c. Downgraded for imprecision: fewer than 300 events in total (optimal information size not met) or a confidence interval that includes both benefit and harm.

d. Downgraded two levels for imprecision: fewer than 20 events in total.

e. By Harbord's and Peters' tests there was no evidence of small-study effects (Egger's test, which is unreliable for sparse binary data, was significant for sepsis only); trim-and-fill imputed three (sepsis) and four (severe complications) studies with adjusted RR 0.57 and 0.56. Not downgraded, because the tests have low power with 11 to 15 informative studies and the adjusted estimates remain below unity.

f. Downgraded for indirectness: in 17 of the 27 contributing studies the count was derived from the events described (reinterventions, intensive care, sepsis, SIRS or septic shock) rather than from Clavien-Dindo grades reported per event.

Supplementary Figure 1. Forest plot for postoperative fever.

Supplementary Figure 2. Forest plot for postoperative sepsis.

Supplementary Figure 3. Forest plot for septic shock.

Supplementary Figure 4. Forest plot for severe postoperative complications (operational definition).

Supplementary Figure 5. Operative time. (A) Forest plot; (B) Baujat plot; (C) leave-one-out sensitivity analysis.

Supplementary Figure 6. Length of hospital stay. (A) Forest plot; (B) Baujat plot; (C) leave-one-out sensitivity analysis.

Supplementary Figure 7. Risk of bias. (A) RoB 2 for the randomized trials; (B) ROBINS-I for the non-randomized studies (low, moderate or serious risk).

Supplementary Figure 8. Forest plot for early residual fragments stratified by tip design of the suction sheath.

Supplementary Figure 9. Forest plot for late residual fragments stratified by tip design of the suction sheath.

Supplementary Figure 10. Contour-enhanced funnel plots. (A) Early residual fragments; (B) late residual fragments; (C) postoperative fever; (D) postoperative sepsis; (E) severe complications; (F) operative time; (G) length of hospital stay. Shaded areas mark the regions of statistical significance at p < 0.10, p < 0.05 and p < 0.01.
